# Dual-MEK Inhibitor Atebimetinib Displays Broad Activity in *RAS*- and *RAF*-Mutant Tumors via Deep Cyclic Inhibition and Resisting RAF Bypass

**DOI:** 10.1158/0008-5472.CAN-25-4907

**Published:** 2026-07-21

**Authors:** Sarah Kolitz, Anna Travesa, Amy Axel, Jason M. Funt, Matthew S. Nord, Amy Yamamura, Kevin D. Fowler, Praveen Nair, Benjamin Zeskind, Brett M. Hall, Peter King

**Affiliations:** 1 Immuneering Corporation, Cambridge, Massachusetts.; 2 Immuneering Corporation, San Diego, California.; 3 Immuneering Corporation, New York, New York.

## Abstract

Inappropriate activation of the mitogen-activated protein kinase (MAPK) pathway, often stemming from activating mutations in *RAS* or *RAF*, represents one of the most common oncogenic events in human cancer. However, currently approved RAS/RAF inhibitors target specific mutants that are only present in a small proportion of RAS- or RAF-activated tumors. Current MEK inhibitors (MEKi) are associated with class-effect toxicities and tumor escape via CRAF bypass of MEK blockade. To address these issues, we designed atebimetinib to resist CRAF-mediated bypass and enable a pharmacokinetic/pharmacodynamic profile termed deep cyclic inhibition (DCI), an approach that transiently but deeply suppresses oncogenic signaling while allowing daily physiologic reset. Unlike chronic or intermittent approaches, DCI optimizes depth and duration of MEK suppression on a daily cycle, blunting rebound signaling while preserving recovery windows for normal tissues. Functioning as an allosteric, selective MEKi, atebimetinib potently inhibited ERK phosphorylation *in vitro* and *in vivo*, resisted CRAF bypass, and was well tolerated and more efficacious in xenograft models of cancer compared with current MEKis. Overall, these findings establish atebimetinib as a dual-MEKi with the potential to provide mutation-agnostic inhibition of the MAPK pathway while leveraging DCI, designed to avoid toxicities associated with existing MEKis. Atebimetinib offers MEK inhibition that is durable and tolerable, supporting the potential of this therapeutic paradigm.

**Significance:**

Atebimetinib, a deep cyclic inhibitor of MEK, blocks MAPK signaling, resists RAF bypass, and is active in *RAS*- and *RAF*-mutant tumors, representing a mechanistically differentiated approach in the field of targeted therapies.

*See related commentary by Gaire and Poulikakos, p. 5017*

## Introduction

Inappropriate activation of the mitogen-activated protein kinase (MAPK) pathway, often stemming from activating mutations in *RAS* or *RAF*, represents one of the most common oncogenic events in human cancer. A total of 3 genes encode 4 distinct RAS proteins: *HRAS, NRAS, *and a splice variant of *KRAS* that gives rise to KRAS4A and KRAS4B. There are 3 RAF proteins: ARAF, BRAF, and RAF1 (CRAF), as well as 2 closely related proteins: kinase suppressor of RAS (KSR) 1 and KSR2. The genes most frequently found to have activating mutations in the RAS, RAF, and KSR families are *KRAS*, *NRAS*, and *BRAF*. Although inhibitors of BRAF and KRAS are approved for clinical use, these work only on specific mutations. Activating mutations of *KRAS* are found in nearly 90% of pancreatic adenocarcinomas, 50% of colorectal adenocarcinomas, and 32% of lung adenocarcinomas (1). The first RAS inhibitors (RASi) developed target the *KRAS* G12C mutation; however, this mutation accounts for only 12% of *KRAS*-mutated solid tumors observed in real-world patients. Other mutations are observed more frequently including the G12D (30%) and G12V (23%) mutations (2). A newer generation of RASi is now in development, targeting a broader range of *KRAS* mutations. In addition, melanomas with activating mutations of *NRAS* (20%) are associated with significantly more aggressive disease, including a greater likelihood of central nervous system involvement and significantly shorter survival times, than those that harbor *BRAF* (47%) mutations or are wild-type (WT) for both *BRAF* and *NRAS* (32%; ref. 3).

In contrast, the mutation targeted by approved RAF inhibitors (RAFi), *BRAF* V600E, is observed in approximately 50% of melanomas (4, 5), and responses to treatment are rapid (6, 7). However, BRAF inhibitor monotherapy drives paradoxical activation of the MAPK pathway in *BRAF* WT cells, which promotes the formation of cutaneous squamous cell carcinoma among other cutaneous abnormalities (8, 9). To counteract these undesirable effects and to improve overall response rates of BRAF inhibitors, they are normally combined with MEK 1 and 2 (MEK) inhibitors (MEKi; ref. 8). Despite the improvements reported with these combinations, grade 3/4 adverse events (AE) are often observed in more than 50% of patients treated with BRAF inhibitor and MEKi combinations. The reported rates of serious AEs (SAE) range from 30% to 40%, and the observed durability of response is limited by acquired resistance, drug dose reductions, and discontinuations (9). The low rates of targetable mutations, association with acquired drug resistance, and high rates of toxicities of these drugs have resulted in an unmet need for new cancer therapeutics that are able to more optimally modulate MAPK signaling in *RAS*- and *RAF*-mutant disease while improving the overall safety profile.

**Figure 1.**
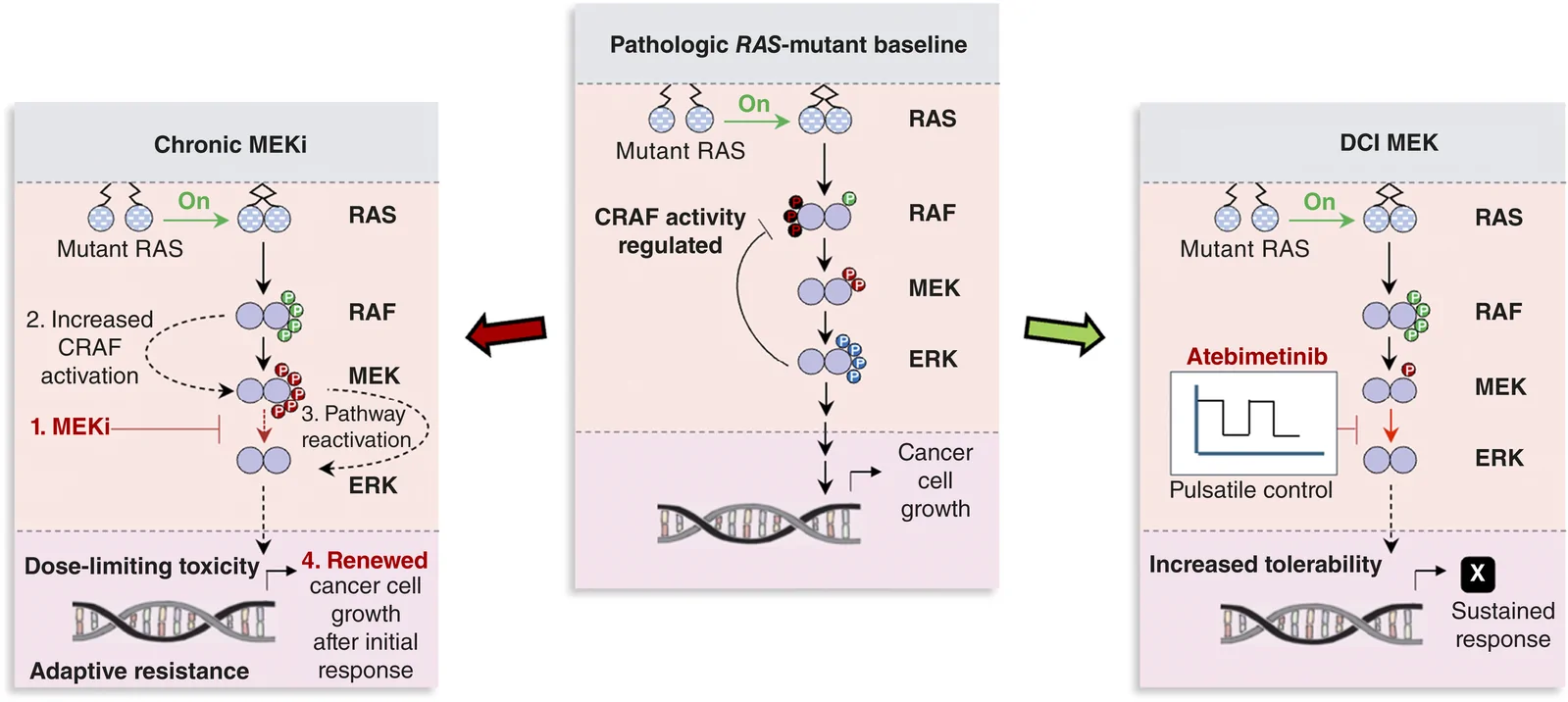
Differences between atebimetinib and chronic MEKis in MAPK pathway control in *RAS*-mutant settings. Middle, normally, RAF (ARAF, BRAF, CRAF, and KSR1/2) activity is allosterically regulated in the presence of pERK. Left, chronic MEKis continuously block the formation of pERK (ERK1/2; 1), thus alleviating CRAF from feedback inhibition and leading to an accumulation of CRAF able to be activated by RAS (HRAS, KRAS, and NRAS; 2). Increasing levels of activated CRAF lead to accumulation of pMEK in the cell, eventually overcoming the blockade by chronic MEKis (3) that, in part, leads to acquired resistance and renewed cancer cell growth (4). Right, atebimetinib is a MEKi that blocks ERK phosphorylation as well as pathway reactivation through CRAF bypass, via DCI. This mechanism of action coupled with a short t_1/2_ leads to sustained responses and increased tolerability.

ERK 1 and 2 (ERK) activity is a key terminal effector of MAPK signaling, and MEK is the “gatekeeper” of ERK activity due to its position in the cascade upstream of ERK and downstream of RAS, RAF, and KSR1 and KSR2 as shown in Fig. 1 (center; refs. 10, 11). In a simplified view of MAPK signaling, RAS is activated either by an environmental signal or as the result of a mutation, and activated RAS promotes homo- or heterodimers of RAF kinases [ARAF, BRAF, or RAF1 (CRAF)] that directly phosphorylate MEK (pMEK). Finally, pMEK activates ERK via phosphorylation (pERK). This process is coordinated and promoted in part by KSR, a scaffold that allosterically activates the RAF kinase domain and brings together RAF, MEK, and ERK to increase signaling efficiency (12). Importantly, the MAPK pathway is tonally regulated by rapid homeostatic feedback via phosphorylation of RAF and KSR to inhibit propagation of MAPK signaling (13, 14). Therefore, MEK represents the single node in the MAPK pathway for processing upstream inputs affecting ERK activation and thus has been an attractive drug target for over 2 decades (15).

Unfortunately, first-generation MEKis have been limited by pathway reactivation events and serious on-target drug toxicities that have restrained clinical utility, especially in patients with *RAS*-mutant tumors (15). Normally, pERK provides homeostatic feedback inhibition of the MAPK pathway by restricting or limiting CRAF activity. However, first-generation MEKis are susceptible to MAPK pathway reactivation in *RAS*-mutant tumors because CRAF activity is less constrained after pERK is depleted by MEK inhibition [Fig. 1 (left)]. Signaling in *RAS*-mutant tumors results in elevated CRAF activity, leading to increased levels of pMEK, which accumulates and eventually overwhelms drug-mediated signaling blockade [Fig. 1 (right); ref. 16]. Therefore, there is a need for MEKis that are not susceptible to CRAF bypass in the setting of tumors with activating *RAS* mutations. Second-generation MEKis aiming to address this issue are under evaluation in the clinic.

In addition, MEKi-associated toxicities occur in part because these drugs have been designed to chronically ablate MAPK signaling, which is normally a dynamic and homeostatic system. In healthy cells, the duration and frequency of ERK activity (i.e., signaling dynamics) are both important in determining the ultimate result, or cell fate, of various signaling events (17). Endogenous ERK signaling is dynamic, with a series of peaks separated by basally active troughs, and different dynamic patterns are produced by different stimuli, which then initiate different responses in downstream transcriptional programs (18). When those patterns are cyclically affected by drugs, threshold changes in cell signaling can drive meaningful cell fate decisions without chronically ablating homeostatic pathways.

**Figure 2.**
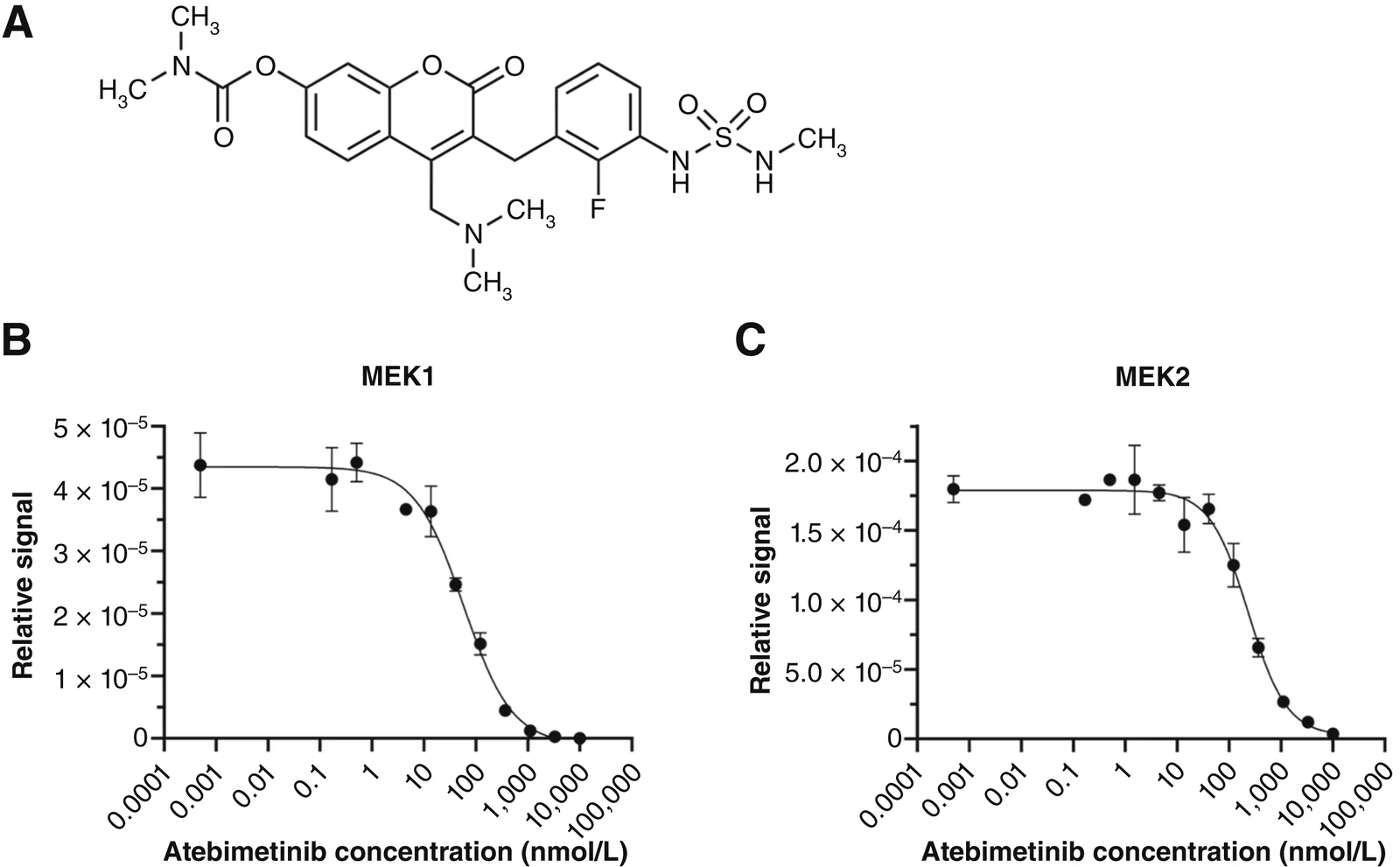
Atebimetinib is an allosteric and selective MEKi with a short residence time. Chemical structure of atebimetinib (**A**). Binding constant (Kd) for atebimetinib was calculated for MEK1 (58 nmol/L; **B**) and MEK2 (237 nmol/L; 2 replicates each; **C**). The amount of kinase measured by qPCR (signal; *y*-axis) is plotted against the corresponding compound concentration in nmol/L in log_10_ scale (*x*-axis).

Atebimetinib (Fig. 2A) is a novel dual-MEKi under clinical evaluation (NCT05585320, NCT07562152), with a new approach to MEK inhibition that is differentiated from first-generation MEKis by allosterically disrupting the activation of MEK itself, instead of inhibiting the ability of MEK to activate ERK alone. A compound with this dual-MEK mechanism should resist CRAF bypass and meaningfully affect tumors with activating mutations of *RAS* or *RAF*. In addition, atebimetinib has a very short half-life (t_1/2_) of 1.2 hours in mice (approximately 2 hours in humans), which allows for chronic dosing while maintaining a near-zero drug trough between doses on a 12- to 24-hour cycle, setting it apart from other second-generation MEKis. This unique pharmacokinetic (PK) profile is expected to improve tolerability by inhibiting MAPK signaling cyclically rather than ablating it chronically. Here, the preclinical attributes of atebimetinib, a dual-MEKi, were assessed showing that its unique pharmacologic profile that combines resistance to feedback mechanisms with a short drug t_1/2_ produces deep cyclic inhibition (DCI) of the MAPK pathway that leads to increased activity and potentially improved safety.

## Materials and Methods

### Experimental model and subject details

#### Cell lines

For *in vitro* experiments, A549 cells (ATCC; cat. #CRM-CCL-185; RRID: CVCL_0023), a human male lung cancer cell line, were cultured in RPMI-1640 medium supplemented with 10% fetal bovine serum (FBS), 2 mmol/L L-glutamine, and 1% penicillin (Pen)/streptomycin (Strep). Cells were maintained as monolayer cultures at 37°C and 5% CO_2_ in humidified air atmosphere, being passaged once/twice weekly at 1 to 5 × 10^6^ cells per T175 flask. For *in vivo* xenograft experiments, A549 tumor cells were maintained *in vitro* with Ham’s F-12K medium supplemented with 10% FBS at 37°C in an atmosphere of 5% CO_2_ in air. The cells in the exponential growth phase were harvested and quantitated using the cell counter before tumor inoculation.

A375 cells (ATCC; cat. #CRL-1619; RRID: CVCL_0132), a human female melanoma cell line with mutated *NRAS*, were maintained in RPMI-1640 media, supplemented with 10% fetal calf serum (FCS), 2 mmol/L L-glutamine, and 1% Pen/Strep for *in vitro* use. Cells were maintained as monolayer cultures at 37°C and 5% CO_2_ in a humidified air atmosphere, being passaged once/twice weekly at 1 to 5 × 10^6^ cells per T175 flask. For *in vivo* xenograft experiments, A375 tumor cells were maintained *in vitro* with DMEM supplemented with 10% FBS at 37°C in an atmosphere of 5% CO_2_ in air. The cells in the exponential growth phase were harvested and quantitated using the cell counter before tumor inoculation.

Colon-26 cells (cat. #0507500-502, DCTD Tumor Repository; RRID: CVCL_0240), a colon cancer cell line derived from BALB/c mice, were maintained in RPMI-1640 media, supplemented with 10% FCS, 2 mmol/L L-glutamine, and 1% Pen/Strep. Cells were maintained as monolayer cultures at 37°C and 5% CO_2_ in a humidified air atmosphere, and cells in the exponential growth phase were harvested and quantitated using the cell counter before tumor inoculation.

NCI-H1299 (ATCC; cat. #CRL-5803; RRID: CVCL_0060) tumor cells were maintained in RPMI-1640 media, supplemented with 10% FCS, 2 mmol/L L-glutamine, and 1% Pen/Strep. Cells were maintained as monolayer cultures at 37°C and 5% CO_2_ in a humidified air atmosphere, being passaged once/twice weekly at 1 to 5 × 10^6^ cells per T175 flask.

SK-MEL-28 (ATCC; cat. #HTB-72; RRID: CVCL_0526) malignant melanoma cells were maintained in RPMI-1640 media, supplemented with 10% FCS, 2 mmol/L L-glutamine, and 1% Pen/Strep. Cells were maintained as monolayer cultures at 37°C and 5% CO_2_ in a humidified air atmosphere, being passaged once/twice weekly at 1 to 5 × 10^6^ cells per T175 flask.

Cell lines were authenticated using IDEXX BioAnalytics CellCheck (which includes a *Mycoplasma* screen), conducted after stocks were initially generated and banked, upon thawing the cell line for experimentation and again after the experiment was completed. Cell lines were kept in culture for ∼2 to 3 months (cell line dependent), with morphology and seeding/harvest densities monitored throughout culturing. Any deviations from the cell line’s established characteristics would result in the cell line being taken down, and the data generated in any experiments at the time of or just prior to changes in the cell line being voided and not used. Specific in-house *Mycoplasma* testing was performed every month [Mycoplasma detection quantitative polymerase chain reaction (qPCR) kit; cat. #TBS42030, Tribioscience].

#### Mice

BALB/c and BALB/c athymic nude mice (Beijing Vital River Laboratory Animal Technology Co., Ltd.; BALB/cAnNCrl, CAnN.Cg-Foxn1nu/Crl; RRID: MGI:2683685) were used. All were female and 7 to 9 weeks at study initiation. Mice were housed at a density of ≤5 per cage at 20°C to 26°C on a 12-hour light/dark cycle with access to standard rodent chow and water *ad libitum*. Mouse studies were reviewed and approved by the Institutional Animal Care and Use Committee of Crown Bioscience.

### Method details

#### KiNativ

A549 lung carcinoma tumor cells (ATCC) were cultured in RPMI-1640 media (Thermo Fisher Scientific) with 10% FBS (Cytiva). Cells were lysed by sonication in a detergent containing lysis buffer and cleared by centrifugation, and the resulting supernatant was collected for compound treatment. The final protein concentration of lysates was 6 mg/mL. Next, 5 µL of atebimetinib was added from 100× stock solutions in dimethyl sulfoxide (DMSO) to 445 µL of lysate in duplicate, so that final compound concentrations were 10 and 1 µmol/L, and 5 µL of DMSO was added to 445 µL of lysate in quadruplicate for controls. After incubation for 15 minutes, 50 µL of a 10× aqueous solution of the desthiobiotin–adenosine triphosphate (ATP)–acylphosphate probe was added to each sample for a final probe concentration of 20 µmol/L and incubated with the samples for 10 minutes. The final DMSO concentration was 1%. Following the probe reaction, samples were prepared for targeted mass spectrometry (MS) analysis using the ActivX standard protocol (19). Briefly, samples were prepared for trypsin digestion (denature and reduce alkylate), digested with trypsin, and desthiobiotinylated peptides enriched on streptavidin resin.

Enriched probe-labeled peptides were analyzed by liquid chromatography/tandem mass spectrometry (LC/MS-MS) on a Thermo-LTQ ion trap mass spectrometer (Thermo Fisher Scientific) using proprietary data collection methodology customized for kinases, as previously described (19). All quantitation was performed by extracting characteristic fragment ion signals from targeted MS/MS spectra and comparing signals in control and treated samples. Proprietary software was used for this purpose with manual validation/visual inspection performed as needed based on data flagging/filtering measures. For each peptide quantitated in this experiment, the change in MS signal for the treated samples relative to the MS signal for the control samples was expressed as percentage inhibition using the following equation:

$$Inhibition\;\left(\%\right)=\left(1-\frac{Average\;MS\;signals\;from\;treated\;samples}{Average\;MS\;signals\;from\;control\;samples}\right)x\;100$$

All inhibited data points were visually verified, as well as all data points showing variability outside of normal limits. The statistical significance of data points showing >35% inhibition was determined according to the Student *t* test (Excel 2010, Microsoft). Scores <0.04 were considered significant:

TTEST(array1,array2,tails,type)

where array1 = MS signals from control samples;
array2 = MS signals from treated samples;
tails = 1 (one-tailed distribution); and
type = 2 (two-sample equal variance).

#### KINOMEscan

The KINOMEscan platform quantitatively measures interactions between test compounds and more than 450 human kinases and disease-relevant mutant variants (20). This technology does not require ATP, and therefore reports thermodynamic interaction affinities, which may or may not have functional relevance to the protein. Kinase-tagged T7 phage strains were grown in parallel in 24-well blocks in an *Escherichia coli* host derived from the BL21 strain. *E. coli* were grown to log-phase and infected with T7 phage from a frozen stock (multiplicity of infection = 0.4) and incubated with shaking at 32°C until lysis (90–150 minutes). The lysates were centrifuged (6,000 × *g*) and filtered (0.2 μm) to remove cell debris. The remaining kinases were produced in HEK-293 cells and subsequently tagged with DNA for qPCR detection. Streptavidin-coated magnetic beads were treated with biotinylated small-molecule ligands for 30 minutes at room temperature to generate affinity resins for kinase assays. The liganded beads were blocked with excess biotin and washed with blocking buffer [SeaBlock (Thermo Fisher Scientific), 1% bovine serum albumin (BSA), 0.05% Tween 20, and 1 mmol/L dithiothreitol (DTT)] to remove unbound ligand and to reduce nonspecific phage binding. Binding reactions were assembled by combining kinases, liganded affinity beads, and test compounds in 1× binding buffer [20% SeaBlock, 0.17× phosphate buffered saline (PBS), 0.05% Tween 20, and 6 mmol/L DTT]. Atebimetinib was prepared as a 40× stock in 100% DMSO and directly diluted into the assay. All reactions were performed in polypropylene 384-well plates in a final volume of 0.02 mL. The assay plates were incubated at room temperature with shaking for 1 hour and the affinity beads washed with wash buffer (1× PBS and 0.05% Tween 20). The beads were then resuspended in elution buffer (1× PBS, 0.05% Tween 20, and 0.5 µmol/L nonbiotinylated affinity ligand) and incubated at room temperature with shaking for 30 minutes. The kinase concentration in the eluates was measured by qPCR. The results for primary screen binding interactions were reported as “% control” using the following equation, in which lower numbers indicate stronger hits in the matrix:

$$\left(\frac{test\;compound\;signal-positive\;control\;signal}{negative\;control\;signal-positive\;control\;signal}\right)\;\times \;100$$

where test compound = compound submitted by Immuneering Corporation;
negative control = DMSO (100% control); and
positive control = control compound (0% control).

#### K_on/off_ methods

##### Kinase protein immobilization

An aliquot of biotinylated kinase protein was thawed, diluted to 40 µg/mL in precooled Biacore buffer (50 mmol/L Tris pH 7.5, 0.05% Tween 20, 150 mmol/L NaCl, 1 mmol/L Tris (2-carboxyl-ethyl)-phosphine-HCl, and 5 mmol/L MgCl_2_) and immobilized with the biotin-tag on a streptavidin (SA) chip. First, the streptavidin on the sensor chip was washed 3 times with a washing solution (1 mol/L NaCl and 50 mmol/L NaOH) for a period of 60 seconds with a flow rate of 10 µL/minute. Then, biotinylated protein was captured on the chip with a targeted immobilization level of 4,000 resonance units (RU). Remaining streptavidin was blocked with 10 µg/mL biocytin injections to circumvent a-specific binding of compounds to the streptavidin-bound surface. To obtain a stable surface with a functional protein, the immobilization procedure was performed at 4°C. Channel 1 was used as a reference channel to correct for buffer effects, whereas other channels were immobilized as described above. After immobilization, the system was buffered with Biacore buffer containing 1% DMSO. After priming 3 times, a prerun was performed for a period of 30 minutes at a flow rate of 30 µL/minute to obtain a stable surface.

##### Single-cycle kinetics assay

To obtain data of good quality, it is important to obtain a stable signal after compound and buffer injection. Therefore, the experiment was started with 5 start-up cycles comprising 5 consecutive injections with buffer performed at 22°C. After the start-up cycles, the signal of the buffer injection is stable, indicating that the surface is ready for compound binding experiments. All compounds were dissolved to a 10 mmol/L solution in 100% DMSO and diluted in Biacore buffer without DMSO to obtain a solution with 1% DMSO. Further dilutions were made in Biacore buffer containing 1% DMSO. This is also used as running buffer during the experiments. In this way, bulk response shifts due to differences in the DMSO concentration are avoided. The single-cycle kinetic experiments were performed with a compound concentration gradient of 1, 3.16, 10, 31.6, and 100 nmol/L for trametinib. For atebimetinib, the concentrations were 10, 31.6, 100, 316, and 1,000 nmol/L. Measurements were performed at a flow rate of 30 µL/minute, a contact time of 100 seconds, and a dissociation period of 1,200 seconds. The single-cycle kinetic experiments were performed at 22°C. A blank run was performed before the compound range was injected. This blank run is an injection range with 5 times a buffer injection with the same flow rate, contact time, and dissociation time. This measurement was subtracted from the compound curve as a double reference to correct for an instable surface.

##### Analysis of the curves

The compound curves were analyzed with Biacore Evaluation Software by using the method of double referencing. In the first step, the reference channel was subtracted from the channel containing immobilized protein, and then the reference curve obtained with buffer injections was subtracted. The resulting curve was fitted with the 1:1 binding model. According to GE HealthCare, the Biacore T200 is designed with the following standard working parameters: association rate constant, ka: 103 to 107 Ms^−1^ and dissociation rate constant, kd: 10^−5^ to 1 s^−1^. This means that for compounds with a very slow dissociation rate (kd < 10^−4^ s^−1^), no exact dissociation constants can be determined. In these cases, Biacore software indicates an inability to fit. For compounds with such extremely slow-off behavior, we report values with operators (> and <) for kd and Kd.

#### K_d_ measurement and calculation

An 11-point 3-fold serial dilution of each test compound was prepared in 100% DMSO at a 100× final test concentration and subsequently diluted to 1× in the assay (final DMSO concentration = 1%). K_d_s were determined using a compound top concentration of 30,000 nmol/L.

Binding constants (K_d_s) were calculated with a standard dose–response curve using the Hill equation. The Hill slope was set to −1. Curves were fitted using a nonlinear least square fit with the Levenberg–Marquardt algorithm.

#### *In vitro* assays

Human tumor cell lines including A549 lung adenocarcinoma (ATCC, cat. #CRM-CCL-185; RRID: CVCL_0023), A375 metastatic melanoma (ATCC, cat. #CRL-1619; RRID: CVCL_0132), NCI-H1299 non–small cell lung cancer (NSCLC; ATCC, cat. #CRL-5803; RRID: CVCL_0060), SK-MEL-28 metastatic melanoma (ATCC, cat. #HTB-72; RRID: CVCL_0526), and a murine colorectal tumor cell line CT-26 (ATCC; cat. #CRL-2638; RRID: CVCL_7254) were used. Tumor cells were maintained in RPMI-1640 media, supplemented with 10% FBS, 2 mmol/L L-glutamine, and 100 U/mL Pen/100 μg/mL Strep. Cells were maintained as monolayer cultures at 37°C and 5% CO_2_ in a humidified air atmosphere, being passaged once/twice weekly at 1 to 5 × 10^6^ cells per T175 flask.

Tumor cells were seeded in 6-well plates at a density of 150,000 cells/well for A549, NCI-H1299, or CT-26, or 120,000 cells/well for A375, or 300,000 cells/well for SK-MEL-28 on day 0. After checking the health and confluence of cells on day 2 (or day 3), media was aspirated and replaced with 1 mL fresh media, or media containing predetermined concentrations of compounds. Cells were further incubated for 2 hours at 37°C and 5% CO_2_ in a humidified air atmosphere. After 2 hours, media was aspirated and cells washed 2 times with cold (4°C) PBS. Cells were lysed (plate on ice) with 50 μL 1× lysis buffer (Cell Signaling Technology) containing 1 mmol/L phenylmethylsulfonylfluoride for 5 minutes. After 5 minutes, cells were removed from the plastic surface using a scraper and transferred to cold 1.5-mL tubes and centrifuged at 14,000 × *g* for 10 minutes at 4°C. The supernatant was gently removed, aliquoted, and snap-frozen in liquid nitrogen. Protein quantification of the lysate was performed with standard Bradford assay using Bradford reagent (Sigma-Aldrich) and analyzed at 595 nm using a SpectraMax M2e plate reader (Molecular Devices). Cell lysates were diluted to 1.0 or 1.5 mg/mL for pERK quantification using the Jess (ProteinSimple) adapted methodology. The following antibodies and dilutions (Ab dilutions optimized) were used for each pERK1/2 (Cell Signaling Technology 4377; 1:50) and tERK1/2 (Cell Signaling Technology 4696; 1:50). All dilutions were made using milk-free antibody diluent.

Levels of phosphoprotein were determined by taking the ratio of phosphorylated protein and the total protein of the target, i.e., phospho-ERK to total ERK, and normalized to the vehicle control. For phospho-ERK and phospho-MEK, phosphorylated protein was detected in the chemiluminescent channel, and total protein was detected in the fluorescent channel. For phospho-CRAF (Ser289/296/301), phosphorylated protein and total protein were both detected in the chemiluminescent channel. Antibodies used for phospho-ERK analysis were phospho-p44/42 MAPK (Erk1/2) (Thr202/Tyr204) (197G2) rabbit monoclonal antibody (mAb; cat. #4377) and p44/42 MAPK (Erk1/2) (L34F12) mouse mAb (cat. #4696). Antibodies used for phospho-MEK analysis were phospho-MEK1/2 (Ser217/221) (41G9) rabbit mAb (cat. #9154) and MEK1/2 (L38C12) mouse mAb (cat. #4694). Antibodies used for phospho-CRAF were phospho-c-Raf (Ser289/296/301) antibody (cat. #9431) and c-Raf (D5X6R) mouse mAb (cat. #12552). All primary antibodies were purchased directly from Cell Signaling Technology. Secondary antibodies for phospho-ERK and phospho-MEK were anti-rabbit secondary horseradish peroxidase [HRP; ProteinSimple (now Bio-Techne), cat. #042-206] and anti-mouse secondary NIR [ProteinSimple (now Bio-Techne), cat. #043-821]. Secondary antibodies used for phospho-CRAF were anti-rabbit secondary HRP [ProteinSimple (now Bio-Techne), cat. #042-206] and anti-mouse secondary HRP [ProteinSimple (now Bio-Techne), cat. #042-205].

#### Three-dimensional Tumor Growth Assays (3D TGA)

Cells were seeded in 3D cultures in a black-walled, clear-bottom, non–tissue culture treated 96-well plate at empirically determined densities (2,500–15,000 cells per well) using a 1:1 mixture of basement membrane extract (BME) and a custom media and grown in a humidified incubator with 5% CO_2_ and 5% O_2_, which approximates capillary levels for each gas, at 37°C. After 24 hours, cells were supplemented with a hormone cocktail made in custom RPMI-1640 media (without phenol red and 1.08 g/L D-glucose pH 6.8–6.9) to achieve final concentrations of 250 pg/mL DHT, 50 pg/mL estradiol (E2), and 1 ng/mL progesterone (P) and were then treated with 9 concentrations of reference compounds or atebimetinib ranging from 10 µmol/L to 1 nmol/L for 72 hours. Ouabain was used at 1 µmol/L as a positive control in select experiments. Cells were assessed for cell proliferation using ethynyl-2′-deoxyuridine (EdU) staining. After 48 hours of treatment, EdU was added to wells to achieve a final concentration of 10 µmol/L. BME plugs were washed twice with PBS, fixed with 3.4% to 3.7% formaldehyde for 20 to 30 minutes at room temperature in the dark, and washed twice with PBS. Cells were permeabilized with 100 µL 0.5% Triton X-100 in PBS for 20 minutes at room temperature. Samples were washed twice gently with PBS. Cells were stained utilizing click-iT chemistry by adding 50 µL of staining cocktail containing 2 mmol/L copper sulfate, 8 µmol/L sulfo-Cy5-azide, and 20 mg/mL ascorbic acid in PBS and incubated for 30 to 60 minutes at room temperature in the dark. Samples were washed 3 times with PBS, stained with 50 µL 4',6-diamidino-2-phenylindole (DAPI) for 30 to 60 minutes at room temperature in the dark, washed twice with PBS, 50 µL PBS added to wells, and kept in dark approximately 48 hours before imaging. Plates were imaged with the Molecular Devices ImageXpress Pico.

EdU data were represented as the percentage of EdU-positive cells quantified by high-content imaging from the Molecular Devices ImageXpress Pico. Best fit curves were generated and IC_50_ values calculated using the R package dr4pl (CRAN, RRID: SCR_003005; ref. 21).

#### PK method

Female BALB/c mice, previously inoculated subcutaneously with Colon-26 tumor cells, were given a single dose of atebimetinib at 12.5, 25, 50, 100, or 150 mg/kg orally. Sparse PK blood and tumor samples were taken at predetermined time points (*n* = 3 per time point) up to 24 hours after dose. Half of samples were processed for plasma and the other half processed for CD3^+^ peripheral blood mononuclear cells. The tumor samples were split into two and were either formalin-fixed paraffin-embedded (FFPE) or snap-frozen (PK). The PK plasma and tumor samples were extracted and analyzed for atebimetinib by LC/MS-MS. Standard noncompartmental analysis of the exposure levels was performed using Phoenix WinNonlin (software version 8.3; RRID: SCR_024504). Composite noncompartmental PK parameter estimates of atebimetinib were calculated. The FFPE tumors were processed for pERK and pMEK using immunohistochemistry (IHC) at Reveal Biosciences. Quantitation digital analysis of pERK1/2 and pMEK IHC was performed using ImageDx software on whole slides, with images being generated by Panoramic Scan (3dhistech; RRID: SCR_024834).

#### Generation of mouse tumor models

To establish a syngeneic and xenograft mouse tumor model, tumor cells in 0.1 mL PBS were implanted subcutaneously into the right flank of 7- to 9-week-old female BALB/c mice (for syngeneic models using Colon-26) or BALB/c athymic nude mice (for xenograft models). After the tumor cells were inoculated, the animals were checked daily for morbidity and mortality. During routine monitoring, the animals were checked for any effects of tumor growth and treatments on behavior such as mobility, food and water consumption, body weight gain/loss (twice per week after randomization), eye/hair matting, and any other abnormalities. Mortality and observed clinical signs were recorded for individual animals in detail.

#### *In vivo* tumor response

Mice were randomized based on the “matched distribution” method into treatment groups (*n* = 8 per group for Colon-26 and *n* = 12 for the rest of the tumor models) including vehicle-treated controls and untreated naïve controls once tumors reached approximately 135 mm^3^ in size. Tumor volumes (TV) were measured twice per week after randomization in 2 dimensions using a caliper, and the volume was expressed in mm^3^ using the formula: V = (L × W × W)/2, where V is the TV, L is the tumor length (the longest tumor dimension), and W is the tumor width (the longest tumor dimension perpendicular to L). Dosing and tumor and body weight measurements were conducted in a laminar flow cabinet. The body weights and TVs were measured using StudyDirector software (version 3.1.399.19; Studylog System, Inc.).

Study treatments were initiated on the day of randomization (day 0) and terminated after 29 days of treatment (day 28). Dosing holidays were used with individual mice that had body weight loss (BWL) > 15%, and dosing resumed when BWL <15%. The mice received supplemental gel when obvious BWL or weakness was observed. The body weight of all animals was monitored throughout the study, and animals were euthanized if they lost >20% of their body weight relative to baseline. All the mice in a treatment group were euthanized if the mean TV of the group was >2,000 mm^3^.

#### Transcriptional effects

RNA sequencing (RNA-seq) was used to examine transcriptional effects of atebimetinib treatment in the KRAS G12D colorectal cancer syngeneic mouse model in tumor and muscle tissue. Transcriptional profiles were assessed after initial treatment and after 18 days of treatment with atebimetinib or selumetinib at 100 mg/kg twice a day. Measurements were taken 2 and 12 hours after the last dose of study treatment. Muscle samples from naïve (non–tumor bearing) mice were also assessed. Tumor and muscle tissues (gastrocnemius/soleus/tibialis anterior pooled per mouse) were collected from the mice and snap-frozen. Total RNA was purified from tumor and muscle samples separately using an RNeasy kit (QIAGEN LLC) according to the manufacturer’s instructions, and the RNA integrity was assessed with an Agilent 2100 bioanalyzer (Agilent) and quantified using the NanoDrop (Thermo Fisher Scientific). Only high-quality RNA samples (optical density (OD)260/280 = 1.8–2.2; OD260/230 ≥ 2.0; RNA integrity number (RIN) ≥ 7, >500 ng) were used to construct sequencing libraries using Illumina TruSeq kits (Illumina Inc.). PolyA mRNA was purified from total RNA using oligo-dT–attached magnetic beads and then fragmented by fragmentation buffer. Taking these short fragments as templates, first-strand cDNA was synthesized using reverse transcriptase and random primers, followed by second-strand cDNA synthesis. Then, the synthesized cDNA was subjected to end-repair, phosphorylation, and “A” base addition according to library construction protocol. Then, sequencing adapters were added to both sides of the cDNA fragments. After PCR amplification for cDNA fragments, the targets of 250 to 350 bp were cleaned up. After library construction, a Qubit double-stranded DNA high sensitivity (HS) assay (Thermo Fisher Scientific) was used to quantify the concentration of the resulting sequencing libraries, whereas the size distribution was analyzed using an Agilent bioanalyzer 2100 (Agilent). After library validation, an Illumina cBot cluster generation system with HiSeq PE Cluster Kits (Illumina) was used to generate clusters. Paired-end sequencing was performed using an Illumina system following Illumina-provided protocols for 2 × 150 paired-end sequencing. A randomization order independent of variables of interest for comparison in the study was utilized for library preparation to help avoid confounding batch effects in the data. All samples were multiplexed in a single sequencing run on the same chip. Raw fastq files were assessed for quality control using fastqc (http://www.bioinformatics.babraham.ac.uk/projects/fastqc/; RRID: SCR_014583), and the results summarized using MultiQC (RRID: SCR_014982; ref. 22). Adapter trimming was performed using bbduk (https://sourceforge.net/projects/bbmap/; RRID: SCR_016969) to remove Illumina universal adapters, and reassessment using fastqc showed successful adapter removal in all samples. Quantification was performed using *Salmon* (RRID: SCR_017036; ref. 23), and MultiQC (RRID: SCR_01498) was used to summarize quality metrics from both fastqc and *Salmon* to produce a report showing quality for all samples after adapter trimming. *Salmon* quantification outputs were processed using the tximport R package (RRID: SCR_016752) to generate a counts matrix. The counts matrix and metadata associated with the samples were used to construct an ExpressionSet (RRID: SCR_014331), which was used for expression quality control and differential expression analysis. Feature filtration was performed to retain samples having at least 20 reads in at least 3 samples. Four samples were removed from analysis because of low median expression (<6). Voom normalization was applied using voomWithQualityWeights. To assess the potential presence of batch effects, associations between sample expression and experimental metadata variables were assessed; principal component analysis and t-distributed stochastic neighbor embedding were also performed to visualize variation in the data. Based on these assessments, no batch correction was performed. The dataset was further assessed for outlier samples using expression z-score distributions as well as intersample distances; 3 samples failed both of these outlier tests and were removed from further analysis. Differential expression analysis was performed using limma-voom (RRID: SCR_010943; ref. 24). Multiple hypothesis correction was performed using the Benjamini–Hochberg method to account for genome-wide assessment. Genes identified as significantly differentially expressed were those genes having an adjusted *P* value <0.05. Differential expression results were assessed for pathway enrichment using gene set enrichment analysis (RRID: SCR_003199; ref. 25).

#### Statistical methods

In the *in vivo* xenograft experiments, the TV of different groups was first compared at a prespecified day using the Bartlett test (26) to check the assumption of homogeneity of variance across all groups. If the *P* value of the Bartlett test was ≥0.05, we ran one-way analysis of variance (ANOVA) to test overall equality of means across all groups. If the *P* value of the one-way ANOVA was <0.05, we further performed *post hoc* analysis using the Tukey honest significant difference test (27) for all pairwise comparisons and Dunnett tests (28) for comparing each treatment group with the vehicle group. When the *P* value of Bartlett test was <0.05, we performed the Kruskal–Wallis test (29) to evaluate overall equality of medians among all groups. If the *P* value of the Kruskal–Wallis test was <0.05, we further performed *post hoc* analysis using the Conover nonparametric test (30) for all pairwise comparisons or for comparing each treatment group with the vehicle group, both with single-step *P* value adjustment. In addition, we performed pairwise comparisons without multiple comparison correction and reported nominal/uncorrected *P* values directly from the Welch *t* test (31) or Mann–Whitney U test (32). Specifically, we first used the Bartlett test to check the assumption of homogeneity of variance for a pair of groups. When the *P* value of the Bartlett test was ≥0.05, we performed the Welch *t* test to obtain nominal *P* values; otherwise, the Mann–Whitney U test was performed. All statistical analyses were done in R: A Language and Environment for Statistical Computing and Graphics, version 3.3.1 (R Project for Statistical Computing, RRID: SCR_001905). All tests were two-sided unless otherwise specified, and *P* values of <0.05 were regarded as statistically significant.

## Results

### Atebimetinib is an allosteric and selective MEKi with a short residence time

Atebimetinib was evaluated with an *in situ* kinome assay (KiNativ) against more than 220 kinases in A549 cells, an NSCLC cell line containing a *KRAS* G12S mutation that encodes a constitutively active protein. This kinome assay is uniquely able to quantitatively profile native protein and lipid kinases based on a proprietary chemoproteomic platform (see Supplementary Methods). The results indicated that atebimetinib selectively inhibited MEK1/2 labeling at the conserved catalytic-domain lysine (Lys1; MEK1 K97 and MEK2 K101), with 72.2% and 94.2% inhibition at 1 and 10 µmol/L, respectively, whereas inhibition at the catalytic-loop lysine (Lys 2; MEK1 K192 and MEK2 K196) was minimal (12.6% and 7.1%, respectively; Supplementary Table S1). KSR1 and KSR2 were inhibited in the assay by 44.7% at the activation loop at 10 µmol/L atebimetinib, a dose higher than the plasma drug free fraction concentrations envisioned for patients but not at 1 µmol/L. All RAF isoforms as well as ERK1/2 did not show significant inhibition by atebimetinib at any of the concentrations tested (Supplementary Table S1).

A thermodynamics-based assay (KINOMEscan) was also used to analyze potential drug–protein interactions of atebimetinib against a panel of 468 WT kinases and disease-relevant mutant variants. The results demonstrate that atebimetinib has high selectivity for MEK1 and MEK2 (95.6% and 92.3% inhibition at 1 µmol/L and 99.9% and 99.6% at 10 µmol/L, respectively; Supplementary Table S2). No other protein showed inhibition higher than 50% except for CRAF with an inhibition of 62% and 66% at 1 and 10 µmol/L, respectively (Supplementary Table S2). This is in contrast to avutometinib, a second-generation MEKi, which has been reported to thermodynamically interact with both BRAF and CRAF (33). However, it should be noted that the KINOMEscan kinase assay was designed and optimized to evaluate ATP-competitive interactions. Therefore, allosteric MEKi interactions identified by this assay may not be functionally consequential.

A follow-up tool of KINOMEscan (KdELECT) was used to calculate the equilibrium dissociation (binding) constants (Kd) for atebimetinib, determined as 58 nmol/L for MEK1 and 237 nmol/L for MEK2 (Fig. 2B and C). Atebimetinib was confirmed to bind MEK1/2 in a non–ATP-competitive manner in an ATP competition assay (Supplementary Fig. S1). In a separate study, the binding kinetics of atebimetinib were evaluated against MEK1 (MAPK2K1) by surface plasmon resonance measurement using Biacore T200 (ResidenceTimer). An association rate constant (ka) of 168,000 M^−1^s^−1^, a dissociation rate constant (kd) of 16.0 ms^−1^, an equilibrium dissociation constant (Kd) of 94.8 nmol/L, t_1/2_ of 43 seconds, and a residence time (t) of 63 seconds were calculated for atebimetinib against MEK1. In contrast, trametinib showed a much longer t_1/2_ and residence time [6,142 and 8,863 seconds, respectively (Supplementary Table S3)].

### Atebimetinib is a potent inhibitor of both pERK and pMEK across *RAS*- and *RAF*-mutant models and is resistant to CRAF bypass

**Figure 3.**
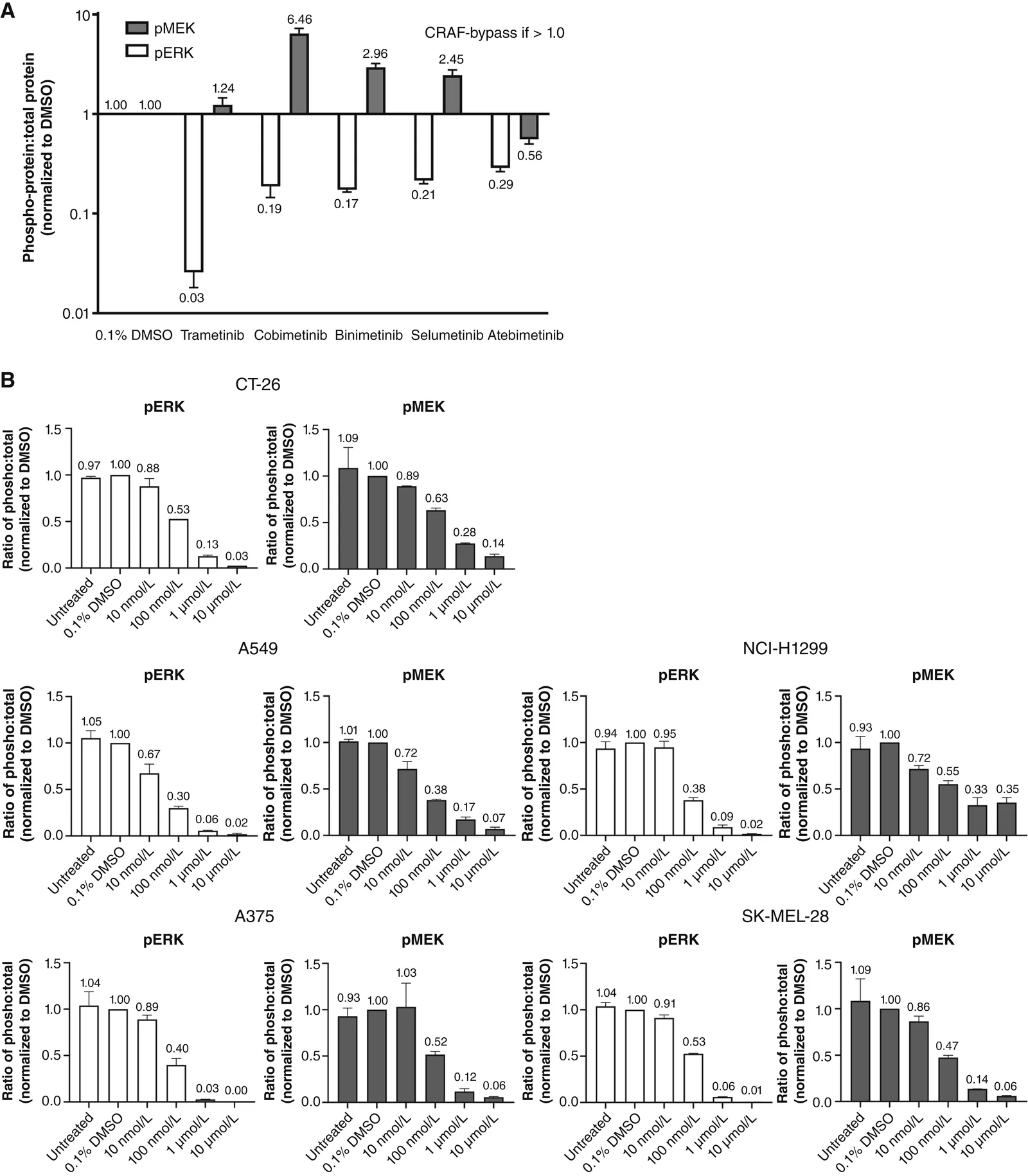
Atebimetinib is a potent inhibitor of both pERK and pMEK across *RAS*- and *RAF*-mutant models and is resistant to CRAF bypass. **A,** Head-to-head comparison of pERK and pMEK levels in A549 *KRAS* G12S–mutant cells treated with atebimetinib and FDA-registered first-generation MEKis at a dose of 100 nmol/L for 2 hours shows the differences in effects on pMEK accumulation, which is a reflection of their susceptibility to pathway bypass by CRAF. **B,** Dual-MEK profile of atebimetinib with 4-point dose–response curves showing decreases in both pERK and pMEK in 5 tumor cell models. Phospho, phosphorylated.

The vulnerability of atebimetinib and other MEKis to CRAF bypass was evaluated in A549 NSCLC cells (*KRAS* G12S; ref. 34) by comparing changes in pERK and pMEK levels following treatment. Digital Western blot analysis showed that pERK was reduced by all inhibitors tested after a 2-hour exposure at a dose of 100 nmol/L (Fig. 3A). However, all 4 approved, first-generation MEKis (trametinib, cobimetinib, binimetinib, and selumetinib) were associated with corresponding increases in cellular pools of pMEK from 1.2- to 6.5-fold over vehicle-treated controls. This result demonstrates that MEK inhibition by these drugs is increasingly susceptible to MAPK pathway reactivation following the initial reduction in cellular pools of pERK. Bypass leads to accumulation of pMEK in the cells as observed and requires first-generation MEKis to sustain target occupancy and maintain drug activity. In contrast, atebimetinib promotes reductions in both pERK and pMEK, illustrating the inability of CRAF bypass or other bypass mechanisms to sustain or increase MEK activation in the presence of this novel inhibitor. This molecular profile allows atebimetinib the opportunity to more effectively modulate the MAPK pathway.

The effect of atebimetinib on MAPK signaling was further assessed using the digital Western blot analysis of pERK and pMEK in a panel of tumor cell lines. Figure 3B shows dose-dependent inhibition of the formation of both pERK and pMEK by atebimetinib across 5 tumor cell models that have activating mutations in *KRAS*, *NRAS*, and *BRAF*. Cell lines tested included CT-26 (murine colorectal carcinoma; *KRAS* G12D), A549 (human NSCLC; *KRAS* G12S), NCI-H1299 (human NSCLC; *NRAS* Q61K), A375 (human melanoma; *BRAF* V600E), and SK-MEL-28 (human melanoma; *BRAF* V600E). A dose-dependent inhibition of pERK was observed in each cell line tested after 2 hours of exposure at concentrations ranging from 10 nmol/L to 10 µmol/L. A 12-point dose–response curve of atebimetinib was used in each of the same 5 models to determine cell-based IC_50_ values for reduction of pERK (pERK IC_50_), which ranged from 24 to 67 nmol/L.

### Atebimetinib has a PK profile that allows for DCI of the MAPK pathway

**Figure 4.**
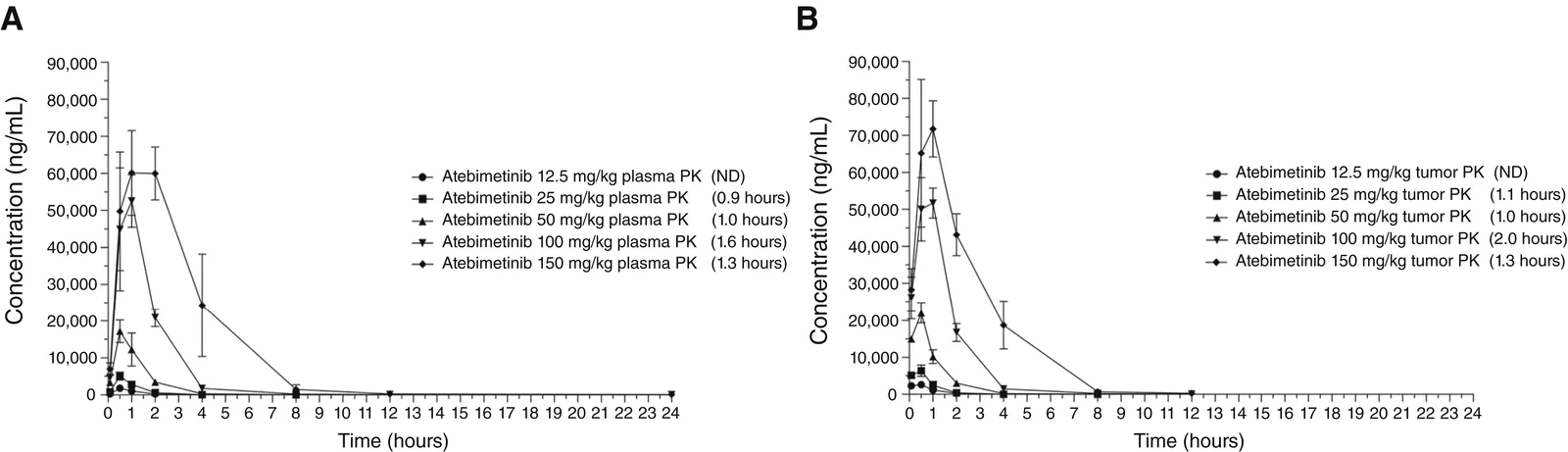
Atebimetinib has a PK profile that allows for DCI of the MAPK pathway. Colon-26 tumor-bearing BALB/c mice were treated with different doses of atebimetinib. PK data from (**A**) plasma and (**B**) tumors in mice demonstrate a short t_1/2_ of atebimetinib of approximately 1.2 to 1.3 hours (based on the average values within plasma and tumor, respectively) as well as effective distribution to the tumor. ND, not determined (i.e., not enough time points after Cmax to allow calculation).

In addition to its novel mechanism of action that is resistant to CRAF bypass, atebimetinib has a short t_1/2_ relative to other MEKis that allows for DCI of the MAPK pathway instead of chronic ablation. Chronic suppression of MAPK signaling is known to drive on-target toxicity for this drug class, but DCI with atebimetinib could improve tolerability (depicted schematically in the graphical abstract). Atebimetinib PK was assessed in the syngeneic Colon-26 (*KRAS* G12D) tumor model in female BALB/c mice treated with a single oral dose of atebimetinib at different concentrations ranging from 12.5 to 150 mg/kg. Atebimetinib levels were then determined over time in both plasma and tumor. Atebimetinib exposure was dose dependent and followed parallel courses of rapid uptake after administration and then a rapid decline to baseline, indicating that plasma concentrations are a good reflection of concentrations in the tumor itself (Fig. 4A and B). In the syngeneic Colon-26 (*KRAS* G12D) tumors in BALB/c mice, the time to maximal plasma concentration (t_max_) was ≤0.5 hours with a plasma t_1/2_ of 1.2 to 1.3 hours (Fig. 4A). Using twice-a-day dosing in mice allows 9.2 to 10 t_1/2_ to pass between doses, so the PK properties of atebimetinib create a profile that achieves fast high-concentration drug levels returning to negligible drug troughs prior to each dosing event.

### Atebimetinib demonstrates DCI through modulation of MAPK pathway pharmacodynamic biomarkers

**Figure 5.**
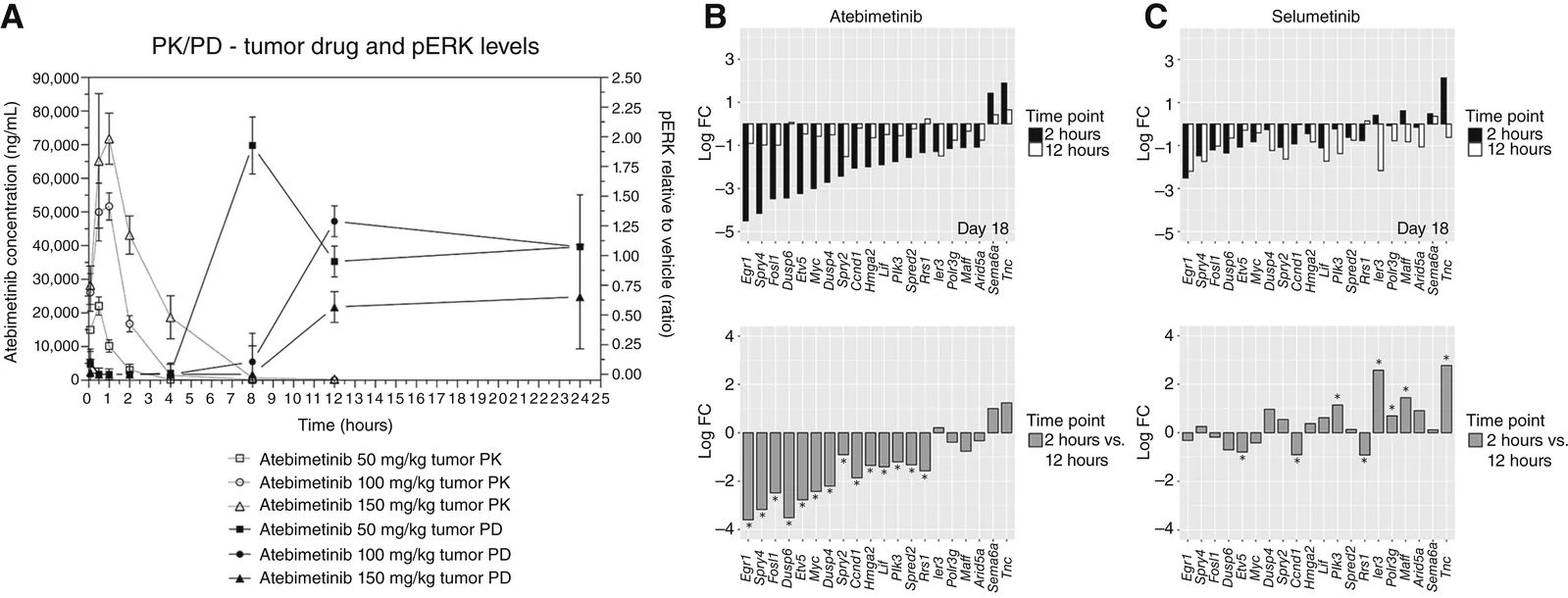
Atebimetinib demonstrates DCI through modulation of MAPK pathway PD biomarkers. **A,** PK (tumor concentration of atebimetinib) and PD (pERK) samples from Colon-26 tumor-bearing BALB/c mice treated with different doses of atebimetinib were quantified at the times indicated. **B,** Differential expression of transcripts for MAPK pathway genes (21) by RNA-seq after 18 days of twice-a-day treatment with atebimetinib showed deep inhibition 2 hours after the last treatment and a return to approximate baseline levels by 12 hours after last dose (top). This DCI pattern is visually summarized (bottom) by the differential expression between transcript levels at 2 and 12 hours following treatment, corrected for vehicle at each time point. FC, fold change. **C,** In contrast, transcript expression for the same set of MAPK pathway genes by RNA-seq after 18 days of twice-a-day treatment with chronic MEKi selumetinib did not show this DCI pattern (top), as is highlighted in visualizing the comparison between the 2- and 12-hour time points, corrected for vehicle at each time point (bottom). Asterisks denote significant (*P* < 0.05) differential expression of that transcript in the vehicle-corrected 2- vs. 12-hour comparison.

Next, we evaluated the pharmacodynamics (PD) of MEK target modulation following oral administration of atebimetinib in the syngeneic Colon-26 tumor model in female BALB/c mice by examining downstream disruption of ERK activation by tumor pERK IHC and MAPK pathway–associated transcriptomic alterations. Evaluation of pERK with IHC was conducted in conjunction with the tumor PK studies. Tumor pERK IHC evaluation was performed in samples from Colon-26 tumor-bearing BALB/c mice that received a single dose of atebimetinib at 50, 100, and 150 mg/kg in an oral suspension and paired to PK drug levels in tumors at multiple time points (*N* = 4 per group). Figure 5A shows the PK/PD relationship between atebimetinib tumor drug level (PK) and level of pERK (PD; see Supplementary Fig. S2 for representative IHC). Atebimetinib sustained near-complete downregulation of pERK for 4 to 8 hours, depending on the oral dose received, followed by a rapid return to endogenous levels after the drug was cleared from the tumor. The PK/PD profile of atebimetinib causes deep inhibition of the MAPK pathway at drug concentration peaks alternating with “near-zero” drug troughs that allow signaling to return to endogenous baseline levels.

Pharmacogenomic studies were used to evaluate evidence for or against DCI following chronic atebimetinib dosing. Tumor-bearing female BALB/c mice were treated orally twice a day with atebimetinib at 100 mg/kg for 18 days. The levels of downstream, ERK-regulated transcripts (35) were assessed by RNA-seq at 2 hours after the final dose, shortly after Cmax, and 12 hours after the final dose of atebimetinib, which aligns with drug trough levels (Fig. 5B). Following the 18-day treatment at twice-a-day dosing, atebimetinib caused dramatic transcriptional changes in most of transcripts assessed 2 hours after dosing followed by a return to near baseline levels by 12 hours after dosing. This illustrates deep cyclic disruption of the MAPK pathway by atebimetinib, which allows for periods of normal, baseline signaling to recover between doses, in contrast to the chronic inhibition produced by earlier-generation MEKis that results in sustaining target engagement through the dosing window by maintaining positive drug troughs (Mektovi. Package insert. Array BioPharma Inc.; 2020; Koselugo. Package insert. AstraZeneca Pharmaceuticals LP; 2020; Cotellic. Package insert. Genentech USA; 2016). Figure 5C shows that the DCI pattern was not observed for MEKi selumetinib.

### Response to atebimetinib in 3D tumor growth assay is driven by the presence of MAPK driver mutation

Atebimetinib was tested across 53 cell lines of diverse indications and mutational profiles in a 3D tumor growth assay (Supplementary Table S4). Cell lines were grouped by response to atebimetinib into sensitive, intermediate, or resistant. As expected, cell lines lacking a driver MAPKmutation tended to be resistant, whereas the majority of models bearing MAPK mutations displayed response to atebimetinib.

### Atebimetinib is highly effective and well tolerated across multiple *RAS*- and *RAF*-mutant tumor models *in vivo*

Finally, we compared the effectiveness and tolerability of atebimetinib across multiple *in vivo* tumor models carrying activating mutations in *KRAS*, *NRAS*, or *BRAF*. We assessed atebimetinib head-to-head against the first-generation, Food and Drug Administration (FDA)–approved MEKi binimetinib. Binimetinib displays the shortest plasma t_1/2_ of any marketed drug in this class at 3.5 hours, is dosed twice a day (Mektovi. Package insert. Array BioPharma Inc; 2020), and is susceptible to MAPK pathway reactivation (Fig. 3B). The animal equivalent dose (AED) of the clinically recommended dose of binimetinib, 45 mg twice a day, is 2.5 mg/kg twice a day orally in mice (Mektovi. Package insert. Array BioPharma Inc; 2020; ref. 36). In contrast, atebimetinib is well tolerated at 100 mg/kg twice a day orally, which generally represents a no-observed AE level in mice. At 150 mg/kg twice a day orally, atebimetinib remains well tolerated and mice sustained baseline body weight within a median of 3% to 5% for up to 4 weeks of chronic twice-a-day dosing (Supplementary Fig. S3). Therefore, dosages of atebimetinib at 100 and 150 mg/kg twice a day orally were highlighted in a head-to-head comparison versus binimetinib, dosed at 3 and 10 mg/kg twice a day orally. The dosage of 3.0 mg/kg twice a day orally results in a human equivalent dose (HED) of approximately 120% of binimetinib’s clinical dose (45 mg twice a day), whereas the dosage of 10 mg/kg twice a day orally has an HED approximately 4-fold higher than the clinical dose (i.e., a hyperdose level).

**Figure 6.**
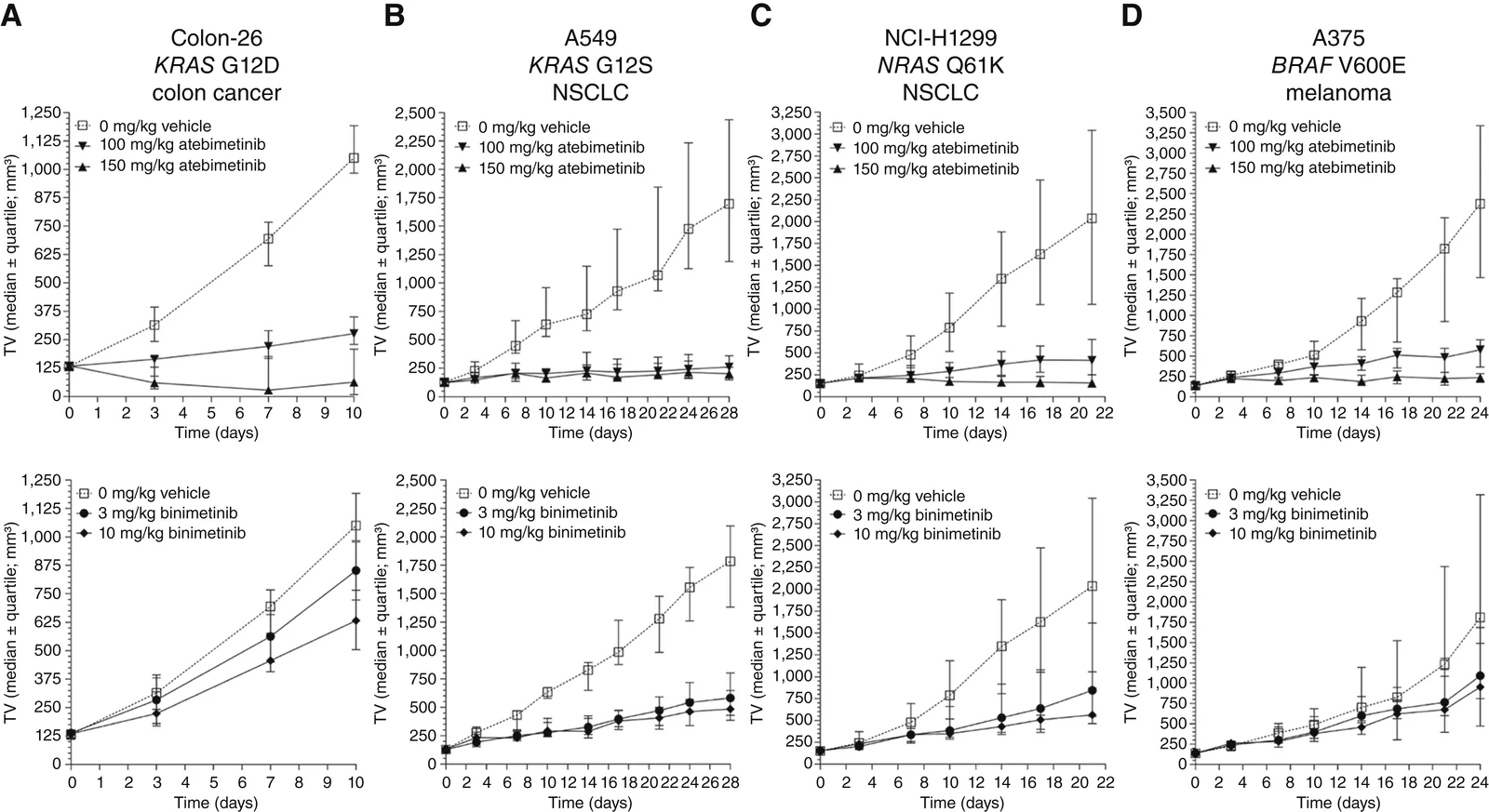
Atebimetinib is highly effective and well tolerated across multiple *RAS*- and *RAF*-mutant tumor models *in vivo*. **A,** Atebimetinib treatment in a Colon-26 *KRAS* G12D colorectal cancer syngeneic model produced TGI results superior to those associated with binimetinib monotherapy (150 mg/kg atebimetinib vs. 10 mg/kg binimetinib: *P* < 0.0001). Results of treatment in xenograft models: **B**, A549, a *KRAS* G12S NSCLC model (150 mg/kg atebimetinib vs. 10 mg/kg binimetinib: *P* < 0.05); **C,** NCI-H1299, an *NRAS* Q61K NSCLC model (150 mg/kg atebimetinib vs. 10 mg/kg binimetinib: *P* < 0.01); and **D**, A375, a *BRAF* V600E melanoma model (150 mg/kg atebimetinib vs. 10 mg/kg binimetinib: *P* < 0.05).

The Colon-26 cell line is widely used in animal models for studying cancer cachexia, leading to rapid BWL in host BALB/c mice (37). Vehicle-treated mice (*N* = 8 per each control and treatment group) succumbed to ethical limits on BWL (more than 20%) after 10 days, which limited our drug treatment comparisons with vehicle and tumor growth inhibition (TGI) calculations. Nevertheless, atebimetinib achieved tumor regressions at 150 mg/kg twice a day orally and a median TGI of 84.3% at 100 mg/kg twice a day orally compared with vehicle-treated mice after 10 days of treatment in the syngeneic model (Fig. 6A). In contrast, the dosages of binimetinib at 3 and 10 mg/kg twice a day orally had limited impact on TV and resulted in TGI of only 22.3% and 45.8%, respectively. The response to the 150 mg/kg dose of atebimetinib was significantly greater than that for the 10 mg/kg hyperdose of binimetinib (*P* < 0.0001). Of note, 5 of the 8 atebimetinib-treated animals displayed near-complete responses within the 10-day treatment window.

We then tested atebimetinib and binimetinib, at the same dose and schedule, in a xenograft model of human *KRAS*-mutated NSCLC by grafting A549 (*KRAS* G12S) cells onto athymic nude (BALB/c) mice. Mice were treated with binimetinib for 28 days at a dose of 3 and 10 mg/kg twice a day orally to approximate AED of the human clinical dose (2.5 mg/kg twice a day orally) and a hyperdose of 4-fold the clinical dose (36). At this elevated dose, binimetinib was able to inhibit tumor growth (Fig. 6B), but the maximum TGI (78.7%) for binimetinib at 10 mg/kg twice a day orally (4-fold HED) was lower than that observed with atebimetinib at either 150 mg/kg twice a day orally (TGI = 95.1%) or 100 mg/kg twice a day orally (91%).

Next, we tested the effectiveness of atebimetinib and binimetinib in human *NRAS*-mutated NSCLC using a xenograft of NCI-H1299 (*NRAS* Q61K) cells in athymic nude mice. Again, both doses of atebimetinib resulted in greater TGI, 86.1% TGI at 100 mg/kg twice a day orally and 99.8% TGI at 150 mg/kg twice a day orally, than either of binimetinib’s doses, namely, 3 mg/kg twice a day orally (TGI = 63.4%) and 10 mg/kg twice a day orally (TGI = 78%; Fig. 6C).

Lastly, we evaluated the activity of atebimetinib in human melanoma with mutated *BRAF* using a xenograft of A375 *BRAF* V600E cells in athymic nude mice. Currently approved treatments for this cancer include BRAF inhibitors in combination with MEKi as MEKi alone has not performed well as single agents in this setting (38). However, a continuing challenge with combinations of MEKi + RAFi is the high frequency of grade 3/4 SAEs, which limits durability, combination options, and clinical utility. Here, we compared atebimetinib head-to-head with binimetinib monotherapy. Consistent with the *RAS*-mutant models described above, atebimetinib produced superior TGIs, 80.4% and 95.6%, when compared with binimetinib, 43.1% and 51.2% (Fig. 6D).

Table 1 summarizes key aspects of all 4 *in vivo* tumor studies, including a full comparison of head-to-head TGIs of binimetinib and atebimetinib, as well as key driver mutation for each model. SK-MEL-28 tumors could not be evaluated using this xenograft model because they grew too slowly to perform *in vivo* pharmacology studies.

**Table 1.** Summary of *in vivo* efficacy studies.

Model (*N* per group)	Tumor type	MAPK mutation	Strain, species, sex	Binimetinib TGI (3 | 10 mg/kg)	Atebimetinib TGI (100 | 150 mg/kg)
Colon-26 (8)	Murine colon carcinoma	*KRAS* G12D	BALB/c, mouse, female	22.3% | 45.8%	84.3% | 107.8%
A549 (12)	Human NSCLC	*KRAS* G12S	BALB/c nude, mouse, female	72.6% | 78.7%	91.3% | 95.1%
NCI-H1299 (12)	Human NSCLC	*NRAS* Q61K	BALB/c nude, mouse, female	63.4% | 78%	86.1% | 99.8%
A375 (12)	Human melanoma	*BRAF* V600E	BALB/c nude, mouse, female	43.1% | 51.2%	80.4% | 95.6%

## Discussion

*RAS* mutations leading to sustained overactivation of the MAPK pathway are common in a variety of human cancers, including pancreatic cancers, NSCLC, colorectal cancer, and melanomas (1–3, 39–42). Although sotorasib has recently been approved as the first mutant RAS inhibitor with others in development, these drugs are not active against the more common, non-G12C oncogenic *RAS* mutations. Notably, approximately 85% of *RAS*-mutant human tumors have *KRAS* mutations, and only 12% to 15% of that subset has the G12C substitution that is targetable by covalent KRAS G12C inhibitors (1). Additionally, 12% of *RAS*-mutant tumors have *NRAS* mutations, most of which harbor mutations at Q61 that are also not susceptible to KRAS G12C covalent inhibitors (1, 3). One strategy to treat tumors with nonaddressable *RAS* mutations is to reverse its downstream effects by targeting the bottleneck of MAPK pathway signaling at MEK. However, MEKis to date have shown poor activity in the context of *RAS*-mutant tumors and are notoriously toxic due to on-target chronic inhibition (15).

**Figure 7.**
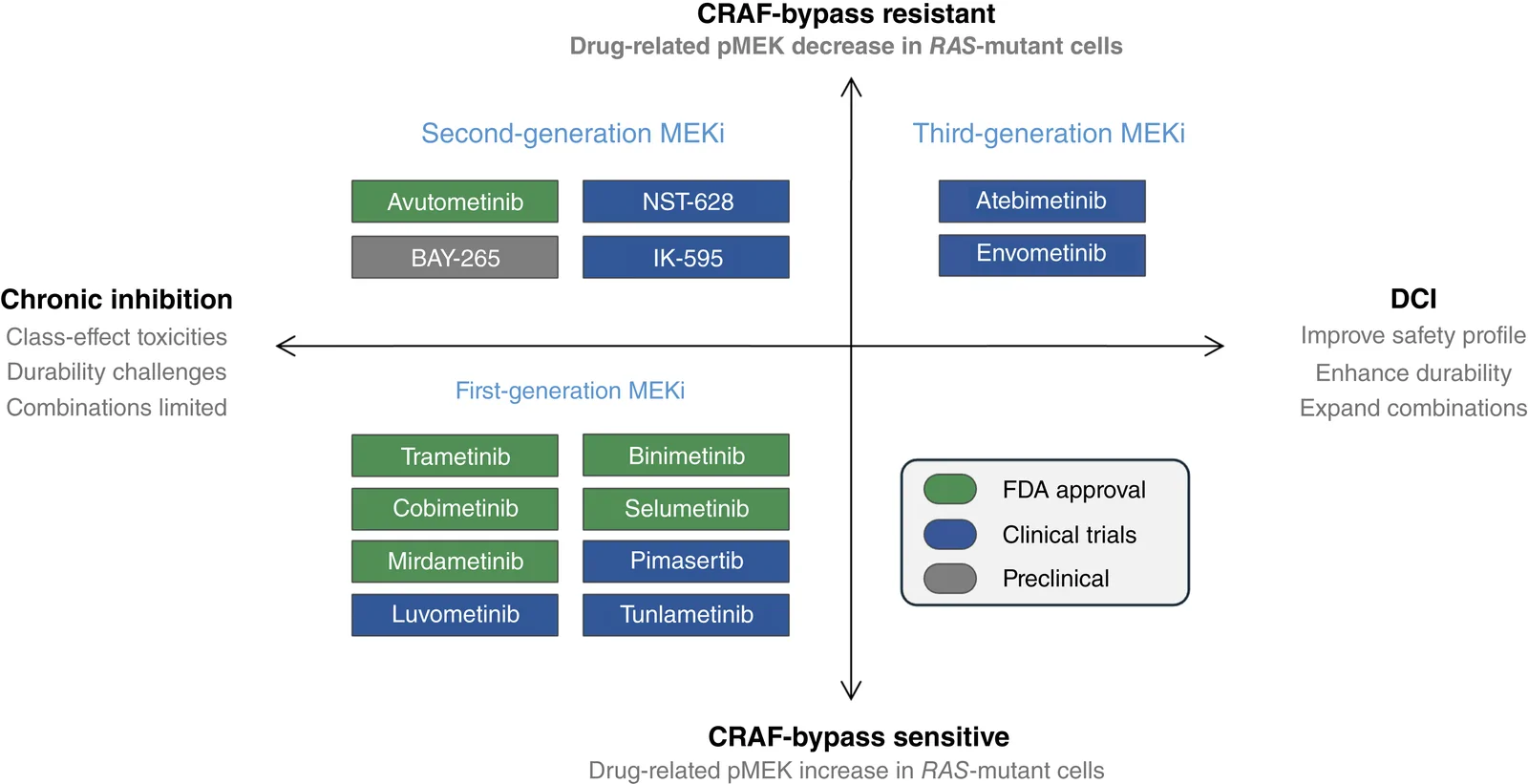
Key attributes of MEKis. Several MEKis that have been approved or are currently in development have been plotted in 2 dimensions: (i) mechanism of action and (ii) toxicity. The mechanism of action dimension ranges from highly susceptible to CRAF bypass (bottom) to highly resistant to CRAF bypass (top). The toxicity dimension ranges from chronic exposure (left) to cyclic exposure (right). In this model, the lower left quadrant contains compounds that are prone to CRAF bypass and associated with higher toxicity, whereas the upper right quadrant includes compounds that are resistant to bypass and associated with lower toxicity. Third-generation MEKis are designed to improve durability and safety by achieving DCI while resisting CRAF bypass.

In general, MEKis can be compared along 2 dimensions, as described in Fig. 7. The first dimension is the duration of MEK inhibition, shaped by both drug exposure (t_1/2_ and dose schedule) and target engagement kinetics (e.g., target residence time). Some MEKis that are approved or in late-stage development have long t_1/2_ leading to chronic MEK inhibition, resulting in greater toxicity and tumor escape, by selecting for tumor cells with lower intrinsic dependence on MAPK pathway signaling. Other MEKis display more moderate plasma t_1/2_, but dosing schedules are more frequent in order to produce similarly chronic MEK inhibition when compared with long-lived MEKis. This also results in greater toxicity and tumor escape.

Preclinical models suggest that DCI is achieved through an elevated plasma drug C_max_ followed by a zero or near-zero drug trough within a 12- to 24-hour window, enabling recovery of MAPK signaling between doses. This requires a minimum of 7 to 8, but no more than 18, drug t_1/2_ between doses. Selumetinib has a human plasma t_1/2_ of 5.3 to 7.2 hours and a t_max_ of 1 to 1.5 hours (43), which results in a sustained, non-zero drug trough with twice-a-day dosing (≤ 2.3 t_1/2_ per twice-a-day cycle) and chronic ablation of MAPK signaling. Binimetinib has a shorter human plasma t_1/2_ of 3.5 hours and t_max_ of 1.6 hours (Mektovi. Package insert. Array BioPharma Inc, 2020), but this still does not allow for a near-zero drug trough (≤ 3.4 t_1/2_ per twice-a-day cycle). Conversely, cobimetinib has a long human plasma t_1/2_ of 44 hours and t_max_ of 2.4 hours [≤ 0.55 t_1/2_ per cycle; Genentech USA (2016)]. Cotellic (cobimetinib; package insert), compared with trametinib, with its long human plasma t_1/2_ of 96 hours has virtually no trough in exposure (≤ 0.25 t_1/2_ per cycle; ref. 44). In contrast, our results indicate that atebimetinib has a very short t_1/2_ (1.2–1.3 hours in rodents and approximately 2 hours in humans) and produces deep but cyclic inhibition of MEK and ERK, leading to improved activity over current chronic MEKis and potentially better safety and durability of response.

DCI is defined not only by systemic exposure kinetics (e.g., t_1/2_ and dosing schedule) but also by the kinetics of target engagement (e.g., potency and target residence time/functional off-rate). In this framework, DCI requires both (i) a short plasma t_1/2_ that enables high peak exposure followed by a near-zero trough within the dosing interval and (ii) sufficiently rapid target disengagement to avoid PD overhang, allowing MAPK signaling to recover between doses in normal tissues. Conversely, “chronic” MEK inhibition can arise from either prolonged exposure (long t_1/2_ and/or frequent dosing) and/or slow target dissociation, each of which favors sustained pathway suppression and can narrow the therapeutic window. This distinction is relevant to interpreting comparisons with slow-offrate, long t_1/2_ MEKis (e.g., trametinib), in which sustained pharmacology is expected to bias toward chronic inhibition even when intermittent dosing is attempted.

The second dimension of MEK inhibition is susceptibility to MAPK pathway reactivation, which includes CRAF bypass, and can be assessed by expanded cellular pools of pMEK in *RAS*-mutant models after exposure to a standard MEKi (16). First-generation MEKis were designed to prevent phosphorylation of ERK by MEK, as a surrogate for pathway activation, by engaging an allosteric pocket adjacent to the ATP-binding pocket of MEK. However, these compounds failed to control MEK phosphorylation on the RAF activation domain, including at Ser218 and Ser222 on MEK1, which is the first step of MEK activation (16). Upon treatment in *RAS*-mutant cells, an increased accumulation of pMEK contributed, in part, to the dogma that MEKis must sustain chronic target occupancy to inhibit MAPK effectively. Chronic MEK inhibition leads to narrow therapeutic windows, high class-effect SAEs, and acquired resistance in tumor cells (15). Consistent with this, Fig. 3 shows that first-generation MEKis suppress pERK while permitting pMEK accumulation (CRAF bypass), whereas atebimetinib suppresses both pERK and pMEK across multiple tumor cell lines.

Unlike current FDA-registered first-generation MEKis, atebimetinib was designed to engage MEK in a way that prioritizes disruption of MEK activation (pMEK) itself. This reduces pMEK as well as pERK in the *RAS*-mutant setting, greatly reducing the potential for MAPK pathway reactivation through CRAF bypass. VS-6766, or avutometinib, a second-generation dual-RAF/MEKi, is also reported to be resistant to CRAF bypass but has a small therapeutic window and higher toxicity due to a long human plasma drug t_1/2_ of approximately 55 to 60 hours, and the issue of low tolerability made intermittent dosing necessary (33). Therefore, there is an unmet need for therapies that reduce signaling through the MAPK pathway that are more tolerable and display more durable effects on tumor growth. Atebimetinib prevents CRAF or other RAF family members from activating MEK and subsequently limits MEK’s ability to phosphorylate or activate ERK, a dual-MEK mechanism. Atebimetinib produces a dose-dependent reduction in the formation of pERK both *in vitro* and *in vivo*. Notably, a parallel reduction in pMEK can also be observed with atebimetinib, demonstrating the basis of resistance to CRAF-mediated bypass in RAS-mutant models. Unlike first-generation MEKis, cellular pools of pMEK do not expand after treatment with atebimetinib.

Atebimetinib, therefore, is a third-generation dual-MEKi that couples a CRAF-mediated bypass resistance with a short t_1/2_ resulting in deep, more durable responses in *RAS*- and *RAF*-mutant tumor models as well as excellent overall tolerability. When compared with other MEKis on the basis of susceptibility to CRAF bypass and chronicity of MAPK pathway suppression (i.e., toxicity), atebimetinib compares favorably with all other compounds on both counts (Fig. 7). This dynamic cyclic inhibition of MEK decouples efficacy and toxicity by allowing MAPK signaling to recover between dosing during cycling near-zero drug troughs. This should lead to improved clinical tolerability as endogenous MAPK signaling is not chronically ablated. This is consistent with observed clinical safety (45). DCI of MAPK pathway also discourages tumor escape by maintaining the tumor’s addiction to MAPK signaling, in contrast to chronic ablation, which encourages development of escape mutations in the target tumor over time. This is aligned with the concept of adaptive therapy (46, 47). The observed tolerability profile would facilitate use in combinations, a key clinical approach both for achieving efficacy and combating resistance.

Here, atebimetinib was effective at reducing tumor growth in several models of *RAS*- and *RAF*-mutated cancers. In models of tumors with *KRAS* mutations that would not likely respond to sotorasib or other G12C RAS inhibitors, atebimetinib was superior to the first-generation MEKis such as binimetinib. *KRAS*-mutated colorectal cancer, represented in our study by Colon-26 (*KRAS* G12D) cells, is not clinically treatable with MEKis. In our syngeneic tumor model, binimetinib was poorly effective, even at 4 times the specified HED, whereas atebimetinib produced a robust tumor response at dosing levels that are well tolerated in preclinical animal models. A549 and NCI-H1299 tumor cells were used to create xenografts of *KRAS* G12S– and *NRAS* Q61K–mutated NSCLC, which do not currently have good clinical treatment options. Although some effect was observed when MEK was inhibited with binimetinib, atebimetinib proved superior in depth and antitumor activity in both models.

We also tested atebimetinib in a model of *BRAF* V600E–mutated melanoma, which has effective, FDA-approved treatments including BRAF inhibitors, in combination with MEKis (8). Atebimetinib was well tolerated and resulted in up to 96% TGI as a monotherapy in this model, and this result was superior to the effectiveness of even high-dose binimetinib monotherapy and was comparable with encorafenib monotherapy at 60 mg/kg every day orally. Toxicity with MEKi alone or in combination with RAFis results in a high frequency of grade 3/4 SAEs, which continue to limit clinical utility, due to sustained patient tolerability, dose reductions, and limited drug–drug combination options (9). Atebimetinib was found to be superior to binimetinib in head-to-head studies, showing the potential for treatment with atebimetinib in *BRAF*-mutant solid tumors as well as *RAS*-mutant solid tumors.

Thus, atebimetinib (IMM-1-104) is a novel, clinically advancing MEKi that integrates a dual-MEK binding mechanism with a chemically optimized scaffold designed for rapid metabolic clearance. This combination enables DCI of the MAPK pathway, an approach that transiently but deeply suppresses oncogenic signaling while allowing daily physiologic reset. Atebimetinib demonstrated broad, mutation-agnostic antitumor activity across diverse *KRAS*-, *NRAS*-, and *BRAF*-mutant models, outperforming first-generation MEKis in both efficacy and durability.

Through comprehensive pharmacologic characterization, we show that atebimetinib’s short plasma t_1/2_ and optimized dosing schedule drive profound pERK suppression during peak exposure (Cmax) while avoiding sustained pathway blockade. Importantly, atebimetinib resists RAF-mediated bypass of MEK inhibition, a key adaptive resistance mechanism that has limited the clinical utility of earlier-generation MEKis.

Together, these findings establish atebimetinib as the first clinically validated example of a DCI-aligned MEKi, offering a new therapeutic paradigm for patients with MAPK-driven tumors. Beyond MEK, the principles of DCI described here may be generalizable to other oncogenic or homeostatic pathways that require transient but potent pharmacologic modulation to optimize therapeutic benefit while preserving normal cellular function.

With clinical development ongoing (NCT05585320, NCT07562152), future efforts will focus on elucidating predictive biomarkers of response, expanding combination strategies with immunotherapies and chemotherapies, and exploring the potential for DCI-based strategies in other kinase-driven malignancies. Atebimetinib represents a mechanistically differentiated approach in the field of targeted therapies, offering the potential to overcome the historical limitations of chronic kinase inhibition.

## Supplementary Material

Supplementary Figure S1 Results of the ATP competition assay with MEK1/2.

Supplementary Figure S2 Example IHC image.

Supplementary Figure S3 Percentage body weight changes in vivo across mouse models.

Supplementary Table S1 Results of an in situ kinome scan (KiNativ) after treating A549 cells with atebimetinib.

Supplementary Table S2 Atebimetinib MEK1/2 specificity in a thermodynamic kinome screen.

Supplementary Table S3 Binding kinetics of atebimetinib and trametinib against MEK1 (MAP2K1).

Supplementary Table S4 Assessment of response to atebimetinib treatment in 53 tumor models in 3D Tumor Growth Assay (3D-TGA).

## Data Availability

There are restrictions in the availability of atebimetinib due to ongoing clinical development. Raw data for this study were generated at Crown Bioscience. Raw RNA-seq data were deposited in the Sequence Read Archive (PRJNA1482191). Data, as well as information and requests for resources and reagents, are available upon request to the corresponding author.
