# Supplementary material for: Dual-MEK Inhibitor Atebimetinib Displays Broad Activity in RAS- and RAF-Mutant Tumors via Deep Cyclic Inhibition and Resisting RAF Bypass
Source: Cancer Res. 2026 Jul 21;86(20):5223–37. doi: 10.1158/0008-5472.CAN-25-4907 (PMC13669731; doi:10.1158/0008-5472.CAN-25-4907)
Supplement: Supplementary file 1 [file can-25-4907_supplementary_figure_s1_suppsf1.docx]

**Supplementary Figure S1. ATP competition assay with MEK1/2.**

The ability of atebimetinib to compete with ATP for binding to MEK1 and MEK2 is shown. The x-axis shows the concentration of atebimetinib added and the enzymatic activity is shown on the y-axis. Atebimetinib is not competitive with ATP binding for either enzyme.
