# Supplementary material for: Dual-MEK Inhibitor Atebimetinib Displays Broad Activity in RAS- and RAF-Mutant Tumors via Deep Cyclic Inhibition and Resisting RAF Bypass
Source: Cancer Res. 2026 Jul 21;86(20):5223–37. doi: 10.1158/0008-5472.CAN-25-4907 (PMC13669731; doi:10.1158/0008-5472.CAN-25-4907)
Supplement: Supplementary file 2 [file can-25-4907_supplementary_figure_s2_suppsf2.docx]

**Supplementary Fig. S2. Example IHC image.**

Supplementary to Fig. 5, examples of pERK1/2-stained sections from Colon-26 tumor bearing mice at 1 and 24h post dose treated with either vehicle or vehicle containing 150 mg/kg atebimetinib. Scale bar represents 0.05mm
