# Supplementary material for: Dual-MEK Inhibitor Atebimetinib Displays Broad Activity in RAS- and RAF-Mutant Tumors via Deep Cyclic Inhibition and Resisting RAF Bypass
Source: Cancer Res. 2026 Jul 21;86(20):5223–37. doi: 10.1158/0008-5472.CAN-25-4907 (PMC13669731; doi:10.1158/0008-5472.CAN-25-4907)
Supplement: Supplementary file 3 [file can-25-4907_supplementary_figure_s3_suppsf3.docx]

D

C

A

B

**Supplementary Fig. S3. Percentage body weight changes *in vivo* in various mouse models*.***

On Day 0, mice were inoculated with a single subcutaneous injection of 0.1 ml of Colon-26 mouse colon carcinoma (A), A549 human non-small cell lung carcinoma (B), NCI-H1299 human non-small cell lung carcinoma (C) and A375 human melanoma (D) cell suspension (5 x 10^5^ cells per animal). Mice (8 per group for Colon-26, 12 per group for A549, NCI-H1299 and A375 tumor bearing mice) were randomized according to tumor volume and treatment started on Day 11, 19, 28 and 14, respectively (day 0 of treatment on graphs). Animals were treated with either vehicle (10% DMSO-90% [20% SBE-β-CD in saline, pH 5.0 ± 0.1] adjusted to pH 3.0 ± 0.1) or vehicle containing atebimetinib or binimetinib at the indicated doses by gavage (PO) for 21, 28, 21 and 28 days, respectively (BID). Body weights were measured twice weekly, and results presented as the percentage body weight change (median mm^3^ ± quartile range %) of each group.
