# Supplementary material for: Dual-MEK Inhibitor Atebimetinib Displays Broad Activity in RAS- and RAF-Mutant Tumors via Deep Cyclic Inhibition and Resisting RAF Bypass
Source: Cancer Res. 2026 Jul 21;86(20):5223–37. doi: 10.1158/0008-5472.CAN-25-4907 (PMC13669731; doi:10.1158/0008-5472.CAN-25-4907)
Supplement: Supplementary file 4 [file can-25-4907_supplementary_table_s1_suppst1.docx]

| **Profile compounds in A549 lysate** | | | | |
| --- | --- | --- | --- | --- |
| **Kinase** | **Reference** | **Labeling Site** | **1 µM atebimetinib** | **10 µM atebimetinib** |
| MAP2K1, MAP2K2 | UniRef100_Q02750, UniRef100_P36507 | Lys1 (K97, K101) | 72.2 | 94.2 |
| KSR1, KSR2 | UniRef100_Q6VAB6, UniRef100_Q8IVT5 | Pseudokinase Domain CA5 (K745, K798) | 23.6 | 44.7 |
| BRAF, CRAF | UniRef100_P04049, UniRef100_P15056 | Activation Loop | 7.9 | 22.1 |
| BRAF, BRAF V600E | UniRef100_P15056_V600E, UniRef100_P15056 | Lys2 | 13.5 | 16.0 |
| MAP2K1 | UniRef100_Q02750 | Lys2 (K192) | 13.2 | 8.6 |
| MAP2K1, MAP2K2 | UniRef100_Q02750, UniRef100_P36507 | Lys2 (K192, K196) | 12.6 | 7.1 |
| CRAF | UniRef100_P04049 | Lys2 | 5.8 | 4.4 |
| Erk1 | UniRef100_P27361 | Lys2 | –7.2 | –2.7 |
| Erk2 | UniRef100_P28482 | Lys2 | –7.0 | –5.4 |
| ARAF | UniRef100_P10398 | Lys2 | –16.2 | –7.0 |
| Percent inhibition of protein kinases by atebimetinib using an *in situ* assay (KiNativ). | | | | |

**Supplementary Table S1.** **Results of an in situ kinome scan (KiNativ) after treating A549 cells with atebimetinib.**

The values in the table reflect percent inhibition in the KiNativ assay (Patricelli, M. P., Nomanbhoy, T. K., Wu, J., Brown, H., Zhou, D., Zhang, J., Jagannathan, S., Aban, A., Okerberg, E., Herring, C., et al. (2011). In situ kinase profiling reveals functionally relevant properties of native kinases. Chem Biol 18, 699-710. 10.1016/j.chembiol.2011.04.011).
