# Supplementary material for: Dual-MEK Inhibitor Atebimetinib Displays Broad Activity in RAS- and RAF-Mutant Tumors via Deep Cyclic Inhibition and Resisting RAF Bypass
Source: Cancer Res. 2026 Jul 21;86(20):5223–37. doi: 10.1158/0008-5472.CAN-25-4907 (PMC13669731; doi:10.1158/0008-5472.CAN-25-4907)
Supplement: Supplementary file 5 [file can-25-4907_supplementary_table_s2_suppst2.docx]

| **Protein Kinase** | **Protein kinase inhibition (%)** | |
| --- | --- | --- |
|  | **1 µM**  **atebimetinib** | **10 µM**  **atebimetinib** |
| MEK1 | 95.6 | 99.9 |
| MEK2 | 92.3 | 99.6 |
| CRAF | 62 | 66 |
| Protein kinases shown to have >50% inhibition by atebimetinib using a thermodynamic assay (KINOMEscan^TM^) against 468 wild type protein kinases. | | |

**Supplementary Table S2.** **Atebimetinib shows MEK1/2 specificity in a thermodynamic kinome screen.**
