# Supplementary material for: Dual-MEK Inhibitor Atebimetinib Displays Broad Activity in RAS- and RAF-Mutant Tumors via Deep Cyclic Inhibition and Resisting RAF Bypass
Source: Cancer Res. 2026 Jul 21;86(20):5223–37. doi: 10.1158/0008-5472.CAN-25-4907 (PMC13669731; doi:10.1158/0008-5472.CAN-25-4907)
Supplement: Supplementary file 6 [file can-25-4907_supplementary_table_s3_suppst3.docx]

| **Compound** | **k_a_ (1/Ms)** | **k_d_ (1/s)** | **K_D_ (M)** | **t_1/2_ (s)** | **T (s)** |
| --- | --- | --- | --- | --- | --- |
| atebimetinib | 1.68E+05 | 1.60E-02 | 9.48E-08 | 43 | 63 |
| trametinib | 1.28E+05 | 1.13E-04 | 8.79E-10 | 6142 | 8863 |
| k_a_: association rate constant; k_d_: dissociation rate constant; K_D_: equilibrium dissociation constant; t_1/2_: half‑life; T: residence time | | | | | |

**Supplementary Table S3.** **Binding kinetics of atebimetinib and trametinib against MEK1 (MAP2K1)**
