# Supplementary material for: Dual-MEK Inhibitor Atebimetinib Displays Broad Activity in RAS- and RAF-Mutant Tumors via Deep Cyclic Inhibition and Resisting RAF Bypass
Source: Cancer Res. 2026 Jul 21;86(20):5223–37. doi: 10.1158/0008-5472.CAN-25-4907 (PMC13669731; doi:10.1158/0008-5472.CAN-25-4907)
Supplement: Supplementary file 7 [file can-25-4907_supplementary_table_s4_suppst4.docx]

| **Cell line** | **RRID** | **Tissue** | **RAS or RAF mutation** | | **Atebimetinib Response in 3D-TGA** | | **p/t ERK** | | | | | | | | **p/t MEK** | | | | | | | |
| --- | --- | --- | --- | --- | --- | --- | --- | --- | --- | --- | --- | --- | --- | --- | --- | --- | --- | --- | --- | --- | --- | --- |
|  |  |  |  |  |  |  | **10uM** | | **1.0 uM** | | **0.10 uM** | | **0.010 uM** | | **10 uM** | | **1.0 uM** | | **0.10 uM** | | **0.010 uM** | |
| MM415 | CVCL_2608 | Melanoma | NRAS:p.Q61L | | Sensitive | | 0.013 | | 0.072 | | 0.336 | | 0.776 | | 0.138 | | 0.324 | | 0.507 | | 1.040 | |
| Hs766T | CVCL_0334 | Pancreatic | KRAS:p.Q61H | | Sensitive | | 0.022 | | 0.061 | | 0.375 | | 0.993 | | 0.121 | | 0.267 | | 0.519 | | 0.950 | |
| NCI-H1299 | CVCL_0060 | Lung | NRAS:p.Q61K | | Sensitive | | 0.016 | | 0.089 | | 0.379 | | 0.948 | | 0.353 | | 0.326 | | 0.552 | | 0.716 | |
| MM127 | CVCL_2601 | Melanoma | NRAS:p.G13R, BRAF:p.G464E:class2 | | Sensitive | | 0.012 | | 0.058 | | 0.243 | | 0.705 | | 0.126 | | 0.248 | | 0.390 | | 0.636 | |
| SK-MEL-2 | CVCL_0069 | Melanoma | NRAS:p.Q61R | | Sensitive | | 0.005 | | 0.027 | | 0.193 | | 0.719 | | 0.392 | | 0.161 | | 0.321 | | 0.684 | |
| MIA PaCa-2 | CVCL_0428 | Pancreatic | KRAS:p.G12C | | Sensitive | | 0.011 | | 0.175 | | 0.277 | | 0.724 | | 0.299 | | 0.575 | | 0.665 | | 1.013 | |
| PA-1 | CVCL_0479 | Ovary | NRAS:p.G12D | | Sensitive | | 0.009 | | 0.082 | | 0.345 | | 0.814 | | 0.104 | | 0.215 | | 0.426 | | 0.822 | |
| SW626 | CVCL_1725 | Ovary | KRAS:p.G12V | | Sensitive | | 0.023 | | 0.065 | | 0.303 | | 0.788 | | 0.167 | | 0.169 | | 0.299 | | 0.732 | |
| MEL-JUSO | CVCL_1403 | Melanoma | HRAS:p.G13D, NRAS:p.Q61L | | Sensitive | | 0.020 | | 0.069 | | 0.313 | | 0.811 | | 0.276 | | 0.377 | | 0.488 | | 0.832 | |
| TT2609-C02 | CVCL_2218 | Thyroid | NRAS:p.Q61R | | Sensitive | | 0.030 | | 0.047 | | 0.171 | | 0.719 | | 0.311 | | 0.277 | | 0.452 | | 0.717 | |
| A375 | CVCL_0132 | Melanoma | BRAF:p.V600E:class1 | | Sensitive | | 0.003 | | 0.026 | | 0.399 | | 0.889 | | 0.056 | | 0.118 | | 0.516 | | 1.031 | |
| RD | CVCL_1649 | eRMS | NRAS:p.Q61H | | Sensitive | | 0.013 | | 0.074 | | 0.255 | | 0.822 | | 0.120 | | 0.221 | | 0.476 | | 0.834 | |
| SK-MEL-28 | CVCL_0526 | Melanoma | BRAF:p.V600E:class1 | | Sensitive | | 0.009 | | 0.061 | | 0.526 | | 0.911 | | 0.059 | | 0.135 | | 0.474 | | 0.864 | |
| Capan-2 | CVCL_0026 | Pancreatic | KRAS:p.G12V | | Intermediate | | 0.016 | | 0.007 | | 0.210 | | 0.683 | | 0.190 | | 0.275 | | 0.444 | | 1.036 | |
| NCI-H358 | CVCL_1559 | Lung | KRAS:p.G12C | | Intermediate | | 0.011 | | 0.040 | | 0.315 | | 0.841 | | 0.140 | | 0.192 | | 0.458 | | 0.833 | |
| SK-MEL-30 | CVCL_0039 | Melanoma | NRAS:p.Q61K | | Intermediate | | 0.024 | | 0.078 | | 0.399 | | 0.839 | | 0.169 | | 0.268 | | 0.628 | | 1.083 | |
| HT-29 | CVCL_0320 | CRC | BRAF:p.V600E:class1 | | Intermediate | | 0.008 | | 0.053 | | 0.488 | | 0.935 | | 0.054 | | 0.112 | | 0.548 | | 0.927 | |
| MeWo | CVCL_0445 | Melanoma |  | | Intermediate | | 0.027 | | 0.104 | | 0.414 | | 0.826 | | 0.359 | | 0.498 | | 0.658 | | 0.830 | |
| COLO 205 | CVCL_0218 | CRC | BRAF:p.V600E:class1 | | Intermediate | | 0.041 | | 0.058 | | 0.270 | | 0.758 | | 0.064 | | 0.123 | | 0.402 | | 0.841 | |
| SNU-475 | CVCL_0497 | Liver |  | | Intermediate | | 0.040 | | 0.238 | | 0.622 | | 0.925 | | 0.338 | | 0.544 | | 0.589 | | 0.795 | |
| SW1463 | CVCL_1718 | CRC | KRAS:p.G12C | | Intermediate | | 0.021 | | 0.058 | | 0.383 | | 0.813 | | 0.250 | | 0.309 | | 0.443 | | 0.863 | |
| AsPC-1 | CVCL_0152 | Pancreatic | KRAS:p.G12 | | Intermediate | | 0.007 | | 0.087 | | 0.480 | | 0.933 | | 0.135 | | 0.201 | | 0.528 | | 0.717 | |
| Hs852.T | CVCL_0950 | Melanoma | NRAS:p.G12V | | Intermediate | | 0.027 | | 0.096 | | 0.400 | | 0.747 | | 0.478 | | 0.475 | | 0.702 | | 1.044 | |
| KATO III | CVCL_0371 | Gastric |  | | Intermediate | | 0.050 | | 0.201 | | 0.470 | | 0.845 | | 0.512 | | 0.649 | | 0.618 | | 0.915 | |
| NCI-H1573 | CVCL_1478 | Lung | KRAS:p.G12A | | Intermediate | | 0.024 | | 0.103 | | 0.361 | | 0.841 | | 0.277 | | 0.320 | | 0.530 | | 0.862 | |
| CFPAC-1 | CVCL_1119 | Pancreatic | KRAS:p.G12V | | Intermediate | | 0.021 | | 0.060 | | 0.212 | | 0.728 | | 0.176 | | 0.312 | | 0.323 | | 0.874 | |
| ASH3 | CVCL_2856 | Thyroid | NRAS:p.Q61R | | Intermediate | | 0.017 | | 0.017 | | 0.131 | | 0.708 | | 0.192 | | 0.216 | | 0.397 | | 0.776 | |
| BxPC3 | CVCL_0186 | Pancreatic |  | | Intermediate | | 0.021 | | 0.055 | | 0.364 | | 0.847 | | 0.139 | | 0.237 | | 0.556 | | 0.914 | |
| SK-N-BE-2 | CVCL_0528 | Neuroblastoma | | Intermediate | | 0.145 | | 0.337 | | 0.543 | | 0.894 | | 1.122 | | 1.307 | | 0.960 | | 1.178 | |  |
| NCI-H1373 | CVCL_1465 | Lung | KRAS:p.G12C | | Intermediate | | 0.019 | | 0.075 | | 0.369 | | 0.631 | | 0.151 | | 0.218 | | 0.472 | | 0.790 | |
| Panc 10.05 | CVCL_1639 | Pancreatic | KRAS:p.G12D | | Intermediate | | 0.014 | | 0.085 | | 0.463 | | 1.220 | | 0.164 | | 0.368 | | 0.556 | | 0.966 | |
| NCI-H1975 | CVCL_1511 | Lung |  | | Intermediate | | 0.016 | | 0.067 | | 0.374 | | 0.836 | | 0.321 | | 0.464 | | 0.790 | | 0.812 | |
| SNU-5 | CVCL_0078 | Gastric |  | | Intermediate | | 0.028 | | 0.091 | | 0.491 | | 0.890 | | 0.115 | | 0.198 | | 0.488 | | 0.791 | |
| SW480 | CVCL_0546 | CRC | KRAS:p.G12V | | Intermediate | | 0.028 | | 0.067 | | 0.275 | | 0.681 | | 0.129 | | 0.217 | | 0.432 | | 0.780 | |
| NCI-H2030 | CVCL_1517 | Lung | KRAS:p.G12C | | Intermediate | | 0.019 | | 0.099 | | 0.413 | | 0.834 | | 0.195 | | 0.267 | | 0.450 | | 0.776 | |
| BT-474 | CVCL_0179 | Breast |  | | Intermediate | | 0.020 | | 0.044 | | 0.261 | | 0.653 | | 0.151 | | 0.281 | | 0.542 | | 0.902 | |
| HCT116 | CVCL_0291 | CRC | KRAS:p.G13D | | Intermediate | | 0.027 | | 0.108 | | 0.408 | | 0.787 | | 0.225 | | 0.314 | | 0.384 | | 0.772 | |
| CAL-12T | CVCL_1105 | Lung | BRAF:p.G466V:class3 | | Intermediate | | 0.009 | | 0.022 | | 0.147 | | 0.889 | | 0.055 | | 0.104 | | 0.225 | | 0.764 | |
| CT-26 | CVCL_7256 | CRC | KRAS:p.G12D | | Intermediate | | 0.025 | | 0.129 | | 0.528 | | 0.879 | | 0.140 | | 0.276 | | 0.632 | | 0.891 | |
| A549 | CVCL_0023 | Lung | KRAS:p.G12S | | Intermediate | | 0.018 | | 0.055 | | 0.300 | | 0.674 | | 0.070 | | 0.172 | | 0.380 | | 0.715 | |
| NCI-H1581 | CVCL_1479 | Lung |  | | Intermediate | | 0.028 | | 0.115 | | 0.562 | | 1.190 | | 0.284 | | 0.566 | | 0.706 | | 1.022 | |
| Capan-1 | CVCL_0237 | Pancreatic | KRAS:p.G12V | | Resistant | | 0.021 | | 0.075 | | 0.339 | | 0.881 | | 0.121 | | 0.225 | | 0.313 | | 0.713 | |
| SW837 | CVCL_1729 | CRC | KRAS:p.G12C | | Resistant | | 0.032 | | 0.145 | | 0.390 | | 0.875 | | 0.382 | | 0.684 | | 0.660 | | 0.857 | |
| HCC38 | CVCL_1267 | Breast |  | | Resistant | | 0.060 | | 0.242 | | 0.622 | | 0.905 | | 0.756 | | 0.692 | | 0.533 | | 0.640 | |
| NCI-N87 | CVCL_1603 | Gastric |  | | Resistant | | 0.040 | | 0.188 | | 0.562 | | 0.852 | | 0.498 | | 0.615 | | 0.618 | | 0.859 | |
| SK-BR-3 | CVCL_0033 | Breast |  | | Resistant | | 0.022 | | 0.153 | | 0.475 | | 0.857 | | 0.601 | | 0.925 | | 1.288 | | 1.267 | |
| SK-N-AS | CVCL_1700 | Neuroblastoma | NRAS:p.Q61K | | Resistant | | 0.049 | | 0.048 | | 0.216 | | 0.778 | | 0.274 | | 0.374 | | 0.561 | | 0.888 | |
| MCF-7 | CVCL_0031 | Breast |  | | Resistant | | 0.082 | | 0.227 | | 0.508 | | 0.813 | | 1.081 | | 0.862 | | 0.750 | | 1.096 | |
| SNU-16 | CVCL_0076 | Gastric |  | | Resistant | | 0.078 | | 0.166 | | 0.605 | | 1.000 | | 0.792 | | 1.061 | | 1.142 | | 1.304 | |
| OVCAR-3 | CVCL_0465 | Ovary |  | | Resistant | | 0.054 | | 0.115 | | 0.389 | | 0.803 | | 0.506 | | 0.477 | | 0.629 | | 0.960 | |
| SKOV-3 | CVCL_0532 | Ovary |  | | Resistant | | 0.037 | | 0.083 | | 0.269 | | 0.643 | | 0.536 | | 0.650 | | 0.743 | | 1.053 | |
| SNU-449 | CVCL_0454 | Liver |  | | Resistant | | 0.024 | | 0.129 | | 0.477 | | 0.975 | | 0.289 | | 0.366 | | 0.537 | | 1.003 | |
| NCI-H1666 | CVCL_1485 | Lung | BRAF:p.G466V:class3 | | Resistant | | 0.017 | | 0.115 | | 0.515 | | 0.864 | | 0.103 | | 0.204 | | 0.458 | | 0.862 | |

p/t = phospho to total ratio; uM = microMolar

Models were considered sensitive if IC50 < 1 uM, intermediate if 1-10 uM or at least 25% inhibition at 10 uM, and resistant otherwise

Onion D, Argent RH, Reece-Smith AM, et al. 3-Dimensional Patient-Derived Lung Cancer Assays Reveal Resistance to Standards-of-Care Promoted by Stromal Cells but Sensitivity to Histone Deacetylase Inhibitors. Mol Cancer Ther. 2016;15(4):753-763. <https://doi.org/10.1158/1535-7163.MCT-15-0598>

**Supplementary Table S4.** **Assessment of response to atebimetinib treatment in 53 tumor models in 3D Tumor Growth Assay (3D-TGA).**

Atebimetinib was tested across cell lines of diverse indications and mutational profiles in a 3D tumor growth assay with demonstrated translational relevance (Onion et al, 2016).
